# Naming the untouchable – environmental sequences and niche partitioning as taxonomical evidence in fungi

**DOI:** 10.1186/s43008-020-00045-9

**Published:** 2020-11-03

**Authors:** Faheema Kalsoom Khan, Kerri Kluting, Jeanette Tångrot, Hector Urbina, Tea Ammunet, Shadi Eshghi Sahraei, Martin Rydén, Martin Ryberg, Anna Rosling

**Affiliations:** 1 Department of Ecology and Genetics, Evolutionary Biology, Uppsala University, Norbyvägen 18D, 752 36 Uppsala, Sweden; 2 Department of Organismal Biology, Systematic Biology, Uppsala University, Norbyvägen 18D, 752 36 Uppsala, Sweden; 3 Department of Molecular Biology, National Bioinformatics Infrastructure Sweden (NBIS), SciLifeLab, Umeå University, Umeå, Sweden; 4 Florida Department of Agriculture and Consumer Services, Division of Plant Industry, Gainesville, Florida 32608 USA

**Keywords:** *Archaeorhizomyces victor nom. seq.*, *Archaeorhizomyces secundus nom. seq.*, Biodiversity, Dark matter fungi/dark taxa, Realized niche, Voucherless taxa

## Abstract

Due to their submerged and cryptic lifestyle, the vast majority of fungal species are difficult to observe and describe morphologically, and many remain known to science only from sequences detected in environmental samples. The lack of practices to delimit and name most fungal species is a staggering limitation to communication and interpretation of ecology and evolution in kingdom *Fungi*. Here, we use environmental sequence data as taxonomical evidence and combine phylogenetic and ecological data to generate and test species hypotheses in the class *Archaeorhizomycetes* (*Taphrinomycotina*, *Ascomycota*). Based on environmental amplicon sequencing from a well-studied Swedish pine forest podzol soil, we generate 68 distinct species hypotheses of *Archaeorhizomycetes*, of which two correspond to the only described species in the class. Nine of the species hypotheses represent 78% of the sequenced *Archaeorhizomycetes* community, and are supported by long read data that form the backbone for delimiting species hypothesis based on phylogenetic branch lengths.

Soil fungal communities are shaped by environmental filtering and competitive exclusion so that closely related species are less likely to co-occur in a niche if adaptive traits are evolutionarily conserved. In soil profiles, distinct vertical horizons represent a testable niche dimension, and we found significantly differential distribution across samples for a well-supported pair of sister species hypotheses. Based on the combination of phylogenetic and ecological evidence, we identify two novel species for which we provide molecular diagnostics and propose names. While environmental sequences cannot be automatically translated to species, they can be used to generate phylogenetically distinct species hypotheses that can be further tested using sequences as ecological evidence. We conclude that in the case of abundantly and frequently observed species, environmental sequences can support species recognition in the absences of physical specimens, while rare taxa remain uncaptured at our sampling and sequencing intensity.

## INTRODUCTION

Species are fundamental units of biodiversity. There is general support for species as entities with their own evolutionary identity and fate, i.e. separately evolving metapopulation lineages, the principle upon which the unified species concept is built (De Queiroz 2007; De Queiroz 1998). Nevertheless, it remains challenging to operationally delimit species. It is increasingly evident that morphology-based taxonomy is not a feasible approach to systematically classify most eukaryotic microorganisms, including fungi (Hibbett 2016) and protists (Keeling and Burki 2019). Diagnostic morphological characters may not be readily available even in some macroscopic animals (Fišer et al. 2018), calling for use of different types of data, including DNA-based methods for delimitation and classification of cryptic species (Dayrat 2005; Padial et al. 2010; Yeates et al. 2011). Often, molecular data play a key role in taxonomy, for example, by utilizing phylogenetic evidence to test hypotheses of species boundaries that were developed using morphological evidence. Over the last decade, DNA-based species delimitation has resolved hundreds of cryptic species, and species diagnosis based solely on DNA sequence data is increasingly accepted in algae (Leliaert et al. 2014). Molecular phylogenetic evidence is also increasingly important, in particular for species delimitation in groups of fungi where it is challenging to identify diagnostic morphological characters (Linde et al. 2017).

Ecological species delimitation is another way of recognizing species (De Queiroz 2007). In nature, niche partitioning is an important process that allows co-existence of species with similar resource requirements (Schoener 1974) such as soil fungal communities (Peay et al. 2008). In accordance with the expectation that closely related species have separate niches in at least one dimension, vertical separation has been demonstrated for sister species of soil fungi as a result of competitive avoidance (Mujic et al. 2016).

Currently, the roughly 140,000 described fungal species are predicted to represent less than one tenth of the true fungal diversity (Hibbett 2016). The submerged lifestyle and microscopic, morphological simplicity of fungi complicates their discovery and formal nomenclature because the physical specimens or images required for naming species in accordance with the International Code of Nomenclature for Algae, Fungi and Plants (ICNafp) can often not be isolated or identified. Many of the undescribed fungi are known from environmental studies using amplicon sequencing (Nilsson et al. 2019a) and are among those referred to as “dark taxa” (Ryberg and Nilsson 2018). Efforts to culture and image “dark taxa” are scarce, and as in the case of the current study, often fruitless, in part because we know so little about these organisms.

One approach to identifying hypothetical species and assigning them an identifier using environmental sequence data is via a cluster-based reference system that includes both environmental and ex-type sequences, for example “virtual taxa” of the MaarjAM database (Öpik et al. 2010) or “species hypotheses” of the UNITE database (Kõljalg et al. 2013; Nilsson et al. 2019b). Such databases are valuable tools for identification of environmental amplicon sequences. However, clustering of often short, single locus sequence data from complex environmental samples cannot replace taxonomy, since outputs are context-dependent and similarity cutoffs remain arbitrary (Ryberg 2015). Alternatives to cluster-based approaches instead use phylogenetic evidence to test which clades are best supported as species based on the difference in expected branch lengths within and between species due to the different processes generating them (Pons et al. 2006; Zhang et al. 2013). These methods do not depend on preset cut-offs, but their success still depends on how distinct the difference in within and between species branch length is, taxon sampling, and potential artifacts in the inferred phylogenetic tree (Ahrens et al. 2016; Fujisawa and Barraclough 2013).

Some researchers cite concerns with the use of environmental sequences from complex samples to detect taxa (Thines et al. 2018; Zamora et al. 2018), partially because sequence variation within OTUs is both biological and artifactual. By including sequence quality data alongside the raw reads, artificial noise can be reduced and amplicon sequence variants (ASVs) can be inferred. Using ASVs instead of sequences representing OTUs can facilitate cross-comparison between studies and make it possible to get an insight into the intra- vs inter- specific sequence variation, which helps resolve species hypotheses.

One particularly enigmatic lineage filled with “dark taxa” is the fungal class *Archaeorhizomycetes* (*Taphrinomycotina*, *Ascomycota*) (Rosling et al. 2011). Sequences of *Archaeorhizomycetes* are frequently observed in environmental DNA samples from soil and roots, and have been detected in more than 100 environmental studies (Hibbett 2016; Menkis et al. 2014). Based on clustering of published environmental amplicon sequences, the class is likely to comprise at least 500 species with a wide geographical distribution, occurring in terrestrial environments around the globe (Menkis et al. 2014). The annotated fungal sequence database UNITE (version 8.0) (Kõljalg et al. 2013) currently has 195 species hypotheses classified as *Archaeorhizomycetes* at the 1.5% dissimilarity threshold. Richness estimates based on clustering at 98.5% of environmental sequences available as Short Read Archive data indicate that *Archaeorhizomycetes* could encompass as many as 16,231 OTUs (Lücking and Hawksworth 2018). Previously known as Soil Clone Group 1 based on environmental sequence data (Porter et al. 2008; Schadt et al. 2003), the first living specimen was isolated into pure culture and validly named in 2011: *Archaeorhizomyces finlayi* Rosling & T. James (Rosling et al. 2011). To date, only one additional species, *Archaeorhizomyces borealis* Menkis, T. James & Rosling (Menkis et al. 2014), has been isolated and described. Even if the species richness estimates based on environmental sequence data are inflated due to sequencing errors or artefacts of OTU clustering methods (Lücking and Hawksworth 2018), the two name-bearing species in the class certainly represent a small fraction of the true diversity. While little is currently known about the ecology and lifestyles of species in the *Archaeorhizomycetes*, studies using environmental sequences indicate that the class is both abundant and diverse in a range of terrestrial ecosystems (Carrino-Kyker et al. 2016; Clemmensen et al. 2015; Cruz-Paredes et al. 2019; Fernández-Martínez et al. 2017; Geml et al. 2014; He et al. 2019; Kluting et al. 2019; Kyaschenko et al. 2017; Levy-Booth et al. 2019; Maghnia et al. 2017; Pinto-Figueroa et al. 2019; Rosling et al. 2013; Sun et al. 2016). In several studies, its abundance is linked to ecological patterns, such as succession and nutrient availability, highlighting the urgent need to recognize species in the class to facilitate communication and identify ecological patterns across studies. Despite extensive culturing efforts, no further cultures of *Archaeorhizomycetes* were successfully isolated in the current study.

To estimate diversity of *Archaeorhizomycetes*, as well as many other under-described fungal lineages, environmental sequence analysis needs to be developed for species hypothesis testing. High abundance in environmental sequence datasets and access to reference sequences for all described species makes *Archaeorhizomycetes* an excellent case to develop novel taxonomic approaches for describing species known only from environmental sequences, without the risk of creating confusion with respect to species lacking description of homologous gene regions. Previous studies have revealed a diverse *Archaeorhizomycetes* community that is both abundant and vertically stratified at our study site (Fransson and Rosling 2014; Lindahl et al. 2007). We combined long and short read ASVs to delimit species hypotheses (SH) in *Archaeorhizomycetes* using the branch length model PTP (Zhang et al. 2013), and test these using ecological species recognition. We hypothesized that sister SHs would have different realized niches, defined as colonization of visually distinct Podzol soil horizons, and tested this using short read relative abundance across soil horizons as a proxy for niche distribution. By combining two lines of evidence, we firmly delimit and describe two novel species.

## MATERIAL AND METHODS

### Field site, sampling, culturing and soil DNA extraction

Soil samples were collected in mid-October 2013 from a pine forest at Ivantjärnsheden field station close to Jädraås (60°49′N, 16°30′E, altitude 185 m) (Persson 1980) (Method S1). Between 1974 and 1990, an experimental field study [experiment Ih2 (9802)] (Axelsson and Bråkenhielm 1980) (Fig. S1) with three different treatments: irrigation and fertilization (IF), irrigation (I), un-manipulated control (UM), and a later clear cut plots (CC) was conducted in the stand. After peeling back the top shrub and moss layer, we collected five soil cores from five locations in each 30 × 30 m plot. Soil cores were separated into visually distinct podzol soil layers: organic soil (O, approximately 0–5 cm depth), mineral elluvial soil (E, 5–8 cm) and mineral illuvial soil (B, 8–15 cm), before pooling the layers for each plot for a total of 36 samples (Table S1). Later, roots were separated from the soil samples for an extensive culturing effort attempting to isolate species in *Archaeorhizomycetes* (Grelet et al. 2010; Menkis et al. 2014; Rosling et al. 2011) (Method S1). We obtained 160 cultures, and successfully sequenced rDNA genes from 98 of these with primers ITS1, ITS4, and LR3 (Gardes and Bruns 1993; Hopple Jr and Vilgalys 1994; White et al. 1990). None of the sequences (deposited in GenBank under accessions MH843963-MH844060) matched *Archaeorhizomycetes*. From the 36 composite soil sample, DNA was extracted using the (Xpedition™ Soil/Fecal DNA miniprep, Zymo Research Corporation, Irvine, California, USA) (Urbina et al. 2016) (Method S2). Two different sequence datasets were generated from these samples: 1) a “phylogenetic” dataset of long amplicons using PacBio SMRT, and 2) an “ecological” dataset based on short read metabarcoding using IonTorrent. The longer reads in the “phylogenetic” sequence dataset provides enough information to resolve deeper nodes in the *Archaeorhizomycetes* tree while the “ecological” dataset provides sufficient sequencing depth and replication to test for species specific realized niches.

#### Generating the “phylogenetic” sequence dataset

Approximately 1,500 bp of the rDNA ITS and LSU region was amplified from all soil DNA extracts using the primer set ITS1F (Gardes and Bruns 1993) and LR5 (Hopple Jr and Vilgalys 1994), with Phusion High-Fidelity DNA polymerase (Thermo Fisher Scientific, Waltham, Massachusetts, USA) (Methods S3). A total of 5, 8 and 3 samples successfully amplified by PCR for O, E and B horizons, respectively (Table S1), and PCR products were pooled separately for the three soil horizons to generate the amplicon libraries (SwO, SwE and SwB) for sequencing at SciLifeLab/NGI (Uppsala, Sweden) on a PacBio RS II system (Pacific Biosciences, Menlo Park, CA, USA). Sequences were delivered to us as error-corrected FASTQ files, and raw reads for the current study are available in ENA (samples ERS3508481-ERS3508483).

The “phylogenetic” sequence dataset was filtered, and long read sequence dataset of all amplified sequence variants (ASVs) was generated using DADA2 (version 1.9.3) (Callahan et al. 2016) (Method S3). Taxonomy was predicted with the ITS2 region using the SINTAX classifier (Edgar 2010) as implemented in VSEARCH (version 2.10.4) (Rognes et al. 2016), and the USEARCH/UTAX reference dataset (version 8.0) (UNITE Community 2019), available from the UNITE database (Kõljalg et al. 2013). This reference dataset was customized by replacing unassigned species level taxonomy with UNITE SH when available.

ASVs belonging to *Archaeorhizomycetes* were identified by aligning the LSU region of the 276 ASVs with the LSU sequences of *A. finlayi* (JF836022), *A. borealis* (KF993708) and the uncultured sister lineage of *Archaeorhizomycetes* GS31 (KY687760) (Tedersoo et al. 2017) using Geneious (version 11.1.4) (Kearse et al. 2012). A maximum likelihood tree using RAxML XSED2 (version 8) using the GTRGAMMA model following recommendations on parametrization in (Kelchner and Thomas 2007), and 1000 iterations for the calculation of bootstrap support (Stamatakis 2014) in the CIPRES portal (Miller et al. 2010). The tree was rooted with two *Rozellomycota* sequences (ASV_229 and ASV_237). Taxonomy predictions with a confidence value of 0.8 or higher using class when available, or else phyla or domain was added to the ASV name using a customized script. Forty-two ASVs representing *Archaeorhizomycetes* were identified as those forming a well-supported clade together with *A. borealis* and *A. finlayi,* and distinct from GS31 (Fig. S2). The corresponding 42 full-length ASVs were aligned in Geneious, and a ML tree was generated in the CIPRES portal as described above. Removing one chimeric sequence resulted in a high quality *Archaeorhizomycetes* rDNA sequence dataset consisting of 41 ASVs ranging in length from 1373 to 1801 bp together representing 52,274 reads (Fig. S2, Table S2, S3). In the end, 272 ASV sequences including assigned taxonomy level with a confidence value of 0.8 or above were published in GBIF (10.15468/8zymuf) and are available in UNITE with accession numbers UDB0779092-UDB0779901.

#### Generating the “ecological” sequence dataset

The ITS2 region of the rDNA genes was amplified using primers gITS7 *forward* (Ihrmark et al. 2012) and modified ITS4m *reverse* (Urbina et al. 2016) using barcoded primers (Table S1) (Method S4). Six PCR products were amplified from each soil sample using a CFR96 Touch™Real/Time PCR Detection system (Bio-Rad Laboratories) as previously described (Urbina et al. 2016). Replicates were combined before purification, and a sequencing library was prepared by pooling maximum 35 ng PCR products from each sample including both negative and positive template controls, loaded onto a 318 chip for PGM Ion Torrent sequencing technology (Life Technologies Corporation, Carlsbad, CA, US), and sequenced at SciLifeLab/NGI (Uppsala, Sweden). The current study was sequenced together with four other studies from different ecosystems for a total of 96 samples on the IonTorrent chip. A total of 4,805,942 raw sequence reads were demultiplexed by the sequencing facility and provided as 96 FASTQ files. Raw reads for the current study are available in ENA (samples ERS4600640-ERS4600675).

The software package DADA2 (version 1.14.0) (Callahan et al. 2016) for R (version 3.6.1) (R Core Team, 2019) was used to quality filter the raw reads and infer ASVs after removing primer sequences with cutadapt (version 2.6 with Python version 3.7.5) (Martin 2011) (Method S4). All 96 samples from the chip were pooled for ASV inference and chimera detection, resulting in a total of 4822 ASVs, representing 1,804,814 reads (37.6% of raw reads). For clarity, ASVs generated from IonTorrent data are called itASVs throughout the text. After removing non-fungal itASVs and itASV_1 (the positive control sequence), the dataset consisted of 4461 itASVs and 1,680,044 reads, of which 282 itASVs and 425,349 reads were putatively *Archaeorhizomycetes* across the sequencing run. The R package vegan (version 2.5–6) (Oksanen et al. 2013) was used to conduct an nMDS ordination of the fungal itASV occurrence matrix across all samples based on per-sample relative abundances using the Bray-Curtis dissimilarity index (Fig. S3). Finally, the ”ecological” dataset was generated by removing all samples from other studies, controls and all itASVs occurring only once across the 36 Jädraås samples. The count-per-sample matrix of the “ecological” dataset covers 1664 itASVs (619,176 reads) (Supplementary datafile 1), and the sequences are published in GenBank (accession numbers MT926458-MT928121). Of these, 123 itASVs (233,667 reads; 37.7% of Jädraås fungal reads) were putatively *Archaeorhizomycetes* (Table S4, Supplementary datafile 2).

#### Delimiting phylogenetic species hypotheses based on environmental sequences

We combined the two sequence datasets described above to delimitate species supported by both phylogenetic and ecological species recognition. In brief, we used PTP to generate species hypotheses (SHs) based on branch length distribution in a ML tree including both long ASVs and itASVs (Zhang et al. 2013). SH distribution across samples was estimated based on combined read counts for all itASVs mapped into a SH. Well-supported sister SHs, i.e. pairs of SHs supported by long read data, were tested for having different realized niches using their relative abundance in the total fungal community. The procedure is specified in Method S5.

#### Placement of local SHs among published *Archaeorhizomycetes* sequences

**Fig. 1:**
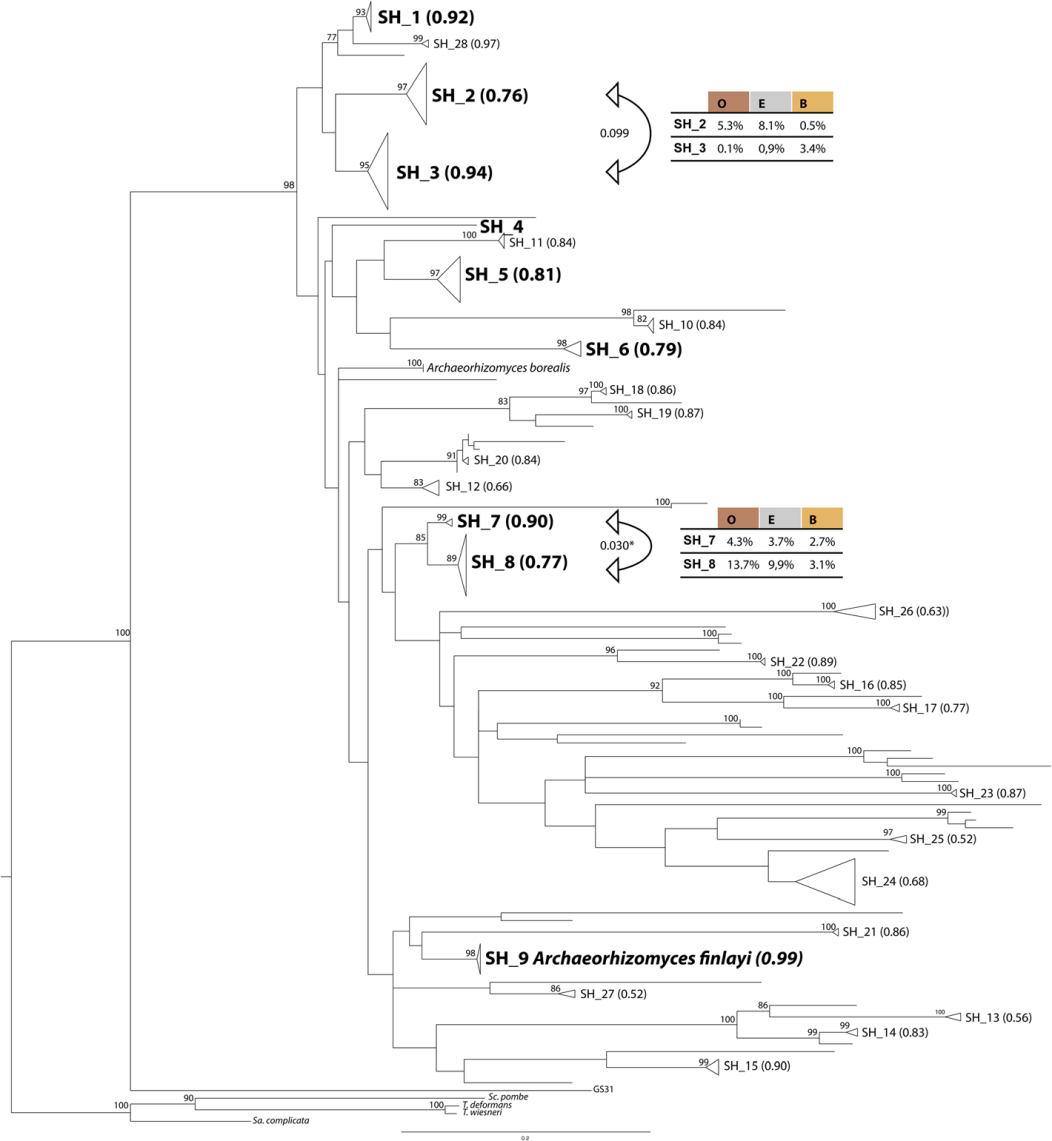
Maximum likelihood phylogenetic inference demonstrating the diversity of *Archaeorhizomycetes* in mid-Sweden pine forest podzol soil based on environmental ribosomal long (41 sequences with ITS and LSU) and short (98 sequences with ITS2) ASVs representing 68 species hypotheses (SH) based on Maximum likelihood solutions in bPTP. Two SHs includes sequences of the described species *Archaeorhizomyces borealis* and *Archaeorhizomyces finlayi*. The tree includes the undescribed sister lineage GS31 (Tedersoo, et al. 2017) and four *Taphrinomycotina* species as outgroup. SHs are cartooned to their stem node to visualize SH represented by more than one ASV. Nine SHs (numbers 1–9) include long read ASVs from the current study and are highlighted in large bold font. Remaining cartooned terminal nodes were represented only by short read itASVs from the current study and are labeled with SH_number or *A. borealis*. Bootstrap support values over 75 are shown on the branches (calculated from 1000 iterations). Average relative sequence read abundance in soil horizons O, E and B are inserted as tables for two pairs of sister SHs. Bent arrows indicate tested orthogonal contrasts with Pr(>F)-values shown in the middle (See Fig. S7a for corresponding tree with all ASV labels displayed).

To place *Archaeorhizomycetes* SHs from the current study in a larger phylogenetic context, an alignment that included publicly available environmental sequences previously identified as belonging to the *Archaeorhizomycetes* (Menkis et al. 2014), as well as new sequences affiliated with the class as identified by BLAST search in UNITE (Altschul et al. 1990), was generated in Geneious (Method S6). Environmental sequences were included if they covered at least two of the three rDNA regions ITS1, ITS2 or LSU. Duplicate sequences from individual studies were excluded. To focus the analysis on SHs from the current study, a series of alignments and ML trees were generated through a process of stepwise removal of published sequences that separated on deep nodes without sequences generated in the current study. The final alignment included a total of 172 *Archaeorhizomycetes* sequences in addition to the 41 ASVs generated in the current study and six outgroup sequences (alignment and tree are available in TreeBASE Study S26320). Global SHs were delimitated across the tree using the bPTP portal and visualized in TreeView by collapsing ML solution SHs as above. All included *Archaeorhizomycetes* sequences were mapped to UNITE species hypotheses at 98.5% by massBLAST of their ITS region (Supplementary datafile 6). The generated tree allowed us to evaluate the robustness of phylogenetic species delimitation in our local dataset and to visualize global sister clade relationships. Further, the larger *Archaeorhizomycetes* alignment was used to visually inspect and identify diagnostic sequences regions in both the ITS1 and ITS2 region for two novel species first hypothesized as SH_7 and SH_8 (Fig. 1).

## RESULTS

Across all samples in the studied pine forest, 33% of the sequenced fungal community was assigned to class *Archaeorhizomycetes* in the short amplicon “ecological” dataset (Table S4). In the “phylogenetic” dataset on the other hand, the class represents 26% of the fungal reads (Table S2). Despite representing a large proportion of the total fungal reads in the dataset (Fig. S4) and intense cultivation efforts, no isolates of *Archaeorhizomycetes* were successfully obtained. We did not detect a significant relationship between the relative abundance of class *Archaeorhizomycetes* and soil horizon (Fig. S5, Table S5) or treatment (Table S5). However, the number of *Archaeorhizomycetes* itASVs and SHs were significantly affected by soil horizon (Table S5), with higher richness detected in B horizon compared to both O and E horizon (Fig. S6). Across all samples, 68 SHs of *Archaeorhizomycetes* were delimited, nine of which were supported by reads in the “phylogenetic” dataset, one of which included the reference sequence of *A. finlayi* (Fig. 1, Fig. S7). The most abundant SHs were supported by long read data in the “phylogenetic” dataset (Fig. S4), together accounting for 78% of the *Archaeorhizomycetes* reads identified in the “ecological” dataset. Likely due to the lower sequencing depth of the “phylogenetic” dataset, taxa making up less than 1% of the sequenced fungal community were not consistently recovered in long read ASVs (Fig. S4).

The *Archaeorhizomycetes* community composition was structured by soil horizons (Fig. S8), and model testing showed that the relative abundance in the “ecological” dataset of nine SHs supported by long reads was significantly affected by soil horizon (PERMANOVA: SH vs Horizon df = 2, *F* = 4.6070, *P* < 0.001) as well as by treatment and plot (Table S6). SH distribution also varied with plot*treatment interactions (df = 3, *F* = 2.0344, *P* > 0.005) (Table S6). Since treatment plots are spatially structured (Fig. S1), we could not separate treatment effects from spatial effects. We conclude that across treatments and plots, soil horizons likely reflect niches explored differently by these fungi. While most of the nine SHs were not significantly associated with a single horizon, we found a marginally significant effect of O horizon on relative abundance of *A. finlayi* (df = 2, χ^2^ = 9.457, *p*_*corrected*_ = 0.792) (Table S7) which was found at highest relative abundance in the B horizons (Fig. S9). For two sister pairs of phylogenetically well-supported SHs that were both abundant and frequently observed (Fig. S4, Table S8), we found significant differences in realized niche for SHs within each pair (Fig. 1, Table S9). Based on their relative sequence read abundance in the fungal community across soil horizons, SH_7 and SH_8 had significantly different niche distributions (Pr(>*F*) = 0.02985). Differential niche distribution of SH_1 and SH_2 was marginally significant (Pr(>*F*) = 0.0995; Table S9). This ecological evidence provides further support for the phylogenetically delimited SH_7 and SH_8 as distinct species with robust boundaries.

### Global perspective on recognized taxa

**Fig. 2:**
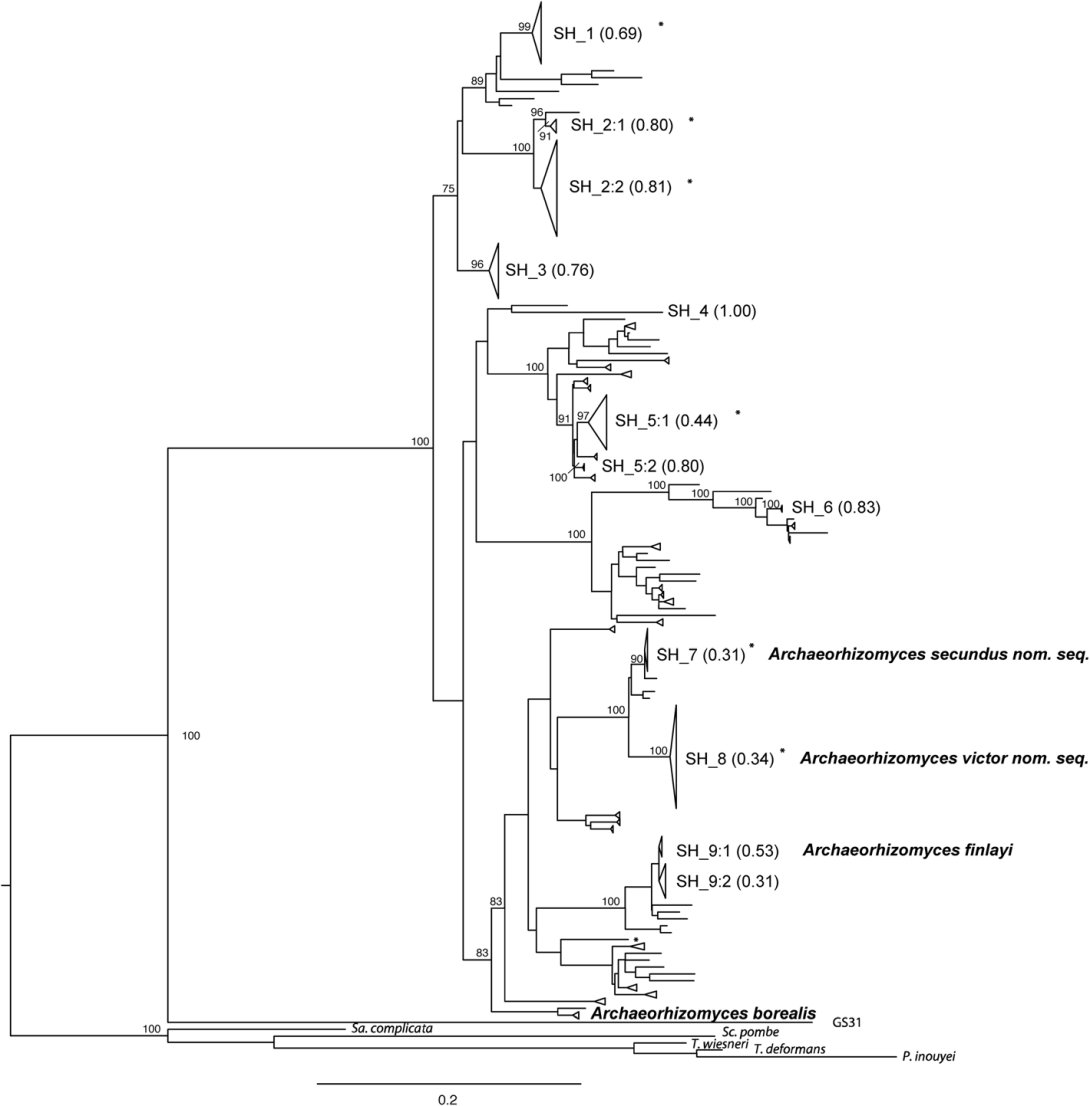
Maximum likelihood tree including all long read ASVs from the current study and publicly available environmental and reference sequences of *Archaeorhizomycetes* covering at least two of the rDNA regions ITS1, ITS2 and LSU. The tree is limited to environmental sequences that cluster on well-supported basal nodes with long read ASV sequences from the current study. Nodes represent 76 species hypothesis (SH) based on Maximum likelihood solutions in bPTP. Nodes including ASVs from the current study are cartooned to visualize how many sequences are included and labeled with SH_number according to Fig. 1, followed by ML support value for species delineation based on bPTP in parenthesis. Names for two described and two novel species are included in bold for their corresponding SHs. Following addition of environmental sequences, three local SHs split, as indicated by adding:1 and:2 after the SH number. SHs including only publicly available sequences are collapsed. Placement of previously published sequences from the studied field station is indicated by *. Bootstrap supports are calculated from 1000 iterations and indicated only when 80 or higher on branches leading up to SH from the current study. Full set of support values and sequence names are available in Fig. S10

The site-specific *Archaeorhizomycetes* diversity (Fig. 1) was analyzed in a global perspective by populating an alignment of ASVs from our “phylogenetic” dataset with publicly available environmental sequences that formed well-supported clades with long read ASVs from *Archaeorhizomycetes* from the current study (Fig. 2, Fig. S10, Supplementary datafile 6). A total of 76 global SHs were delimited and supported using a PTP model, 41 of which are represented by a single sequence (Fig. 2). However, some of the SHs delimited in the local dataset (Fig. 1) were not stable after the removal of short read itASVs and the addition of published environmental sequences. Most notably, SH_2, SH_5 and SH_9 (identified as *A. finlayi*) each split in the global ML tree (Fig. 2). SH_2 separates into two global SHs, both containing sequences previously recovered from the studied field site, with SH_2:2 being the more frequently observed of the two and including long sequences in UNITE SH1566367.08FU, while sequences in SH 2:1 did not map to any existing UNITE SHs (Supplementary datafile 6). SH_5 also separates into two global SHs, with SH_5:1 containing previously published sequences from our field site, as well as sequences from Ireland and the US, while global SH_5:2 contained only two ASVs from the current study (Supplementary datafile 6). Both global SH_5:1 and SH_5:2 cluster on a well-supported branch with four global SHs that include previously published sequences (Fig. 2). Sequences in three of these SHs map to the same UNITE SH (SH1571336.08FU), demonstrating that the boundaries of phylogenetically delimited SHs and cluster-based SHs are not always the same. Further, ASVs and published sequences mapping to SH1556760.08FU, *A. finlayi*, split into two global SHs (SH_9:1 and SH_9:2) in the current analysis (Fig. 2, Supplementary datafile 6). These two SHs cluster on a well-supported branch with five single sequence SHs mapping to four different UNITE SHs. Limited taxon sampling and low representation of intra-species genetic variation of potential sister taxa as well as possible chimeras among published sequences within the clade may obscure phylogenetic SH delimitation.

The included sequences represent only a fraction of the global *Archaeorhizomycetes* diversity, including sequences from 90 of the 181 UNITE SH currently identified as belonging to *Archaeorhizomycetes* (Kõljalg et al. 2013). With the exception of SH_4 and SH_2:1, long read ASVs mapped to UNITE SH, meaning that the majority of our ASVs were highly similar to previously observed environmental sequences (Supplementary datafile 6). *Archaeorhizomyces borealis* is distributed across the Eurasian boreal biome (Menkis et al. 2014) but was rare at our study site and was only detected as a short amplicon itASV.

In the global analysis, the sister taxa SH_7 and SH_8 cluster together with three single sequences (Fig. 2). One of these (GenBank Acc nr: JN006470) represents sequences from UK and Germany (Cox et al. 2010) and clusters closely to SH_7. This sequence maps to UNITE SH1566370.08FU, together with sequences in SH_7 (Supplementary datafile 6). The other two sequences are from Canada and the US and map to UNITE SH1566388.08FU (Supplementary datafile 6). Based on the GlobalFungi database (globalfungi.com) (Vetrovsky et al. 2020) the later SH is restricted to North America. Together, these observations indicate that closely related taxa exist globally, but that these could be geographically separated from those captured in our dataset. While addition of publicly available environmental sequences weakened the PTP ML support for these two SHs, both remain intact. In the global tree, the delimitation of SH_7 and SH_8 both include sequences previously recovered from the studied field site as well as sequences collected throughout Europe (Supplementary datafile 7). These two SHs are distinct based on both local (Fig. 1) and global (Fig. 2) phylogenetic evidence, as well as ecological evidence, since they have significantly different realized niches across samples in the studied Podzol profile (Fig. S9, Table S9). Overall, relative abundance of SH_8 is higher (8.9 ± 1.7%) across all three horizons compared to SH_7 (3.5 ± 1.2%). Relative abundance of SH_8 is highest in O horizon and decreases towards deeper mineral soil layers while the relative abundance of PH_7 is stable throughout the soil profile (Fig. S9b). SH_8 tends to represent a larger proportion of the sequenced fungal community compared to SH_7 when the two are detected together, high relative abundance of SH_7 is restricted to when the relative abundance of SH_8 is low (Fig. S9c) indicating competitive avoidance between the two species similar to patterns of vertical separation previously observed for soil fungal sister species (Mujic et al. 2016).

**Fig. 3:**
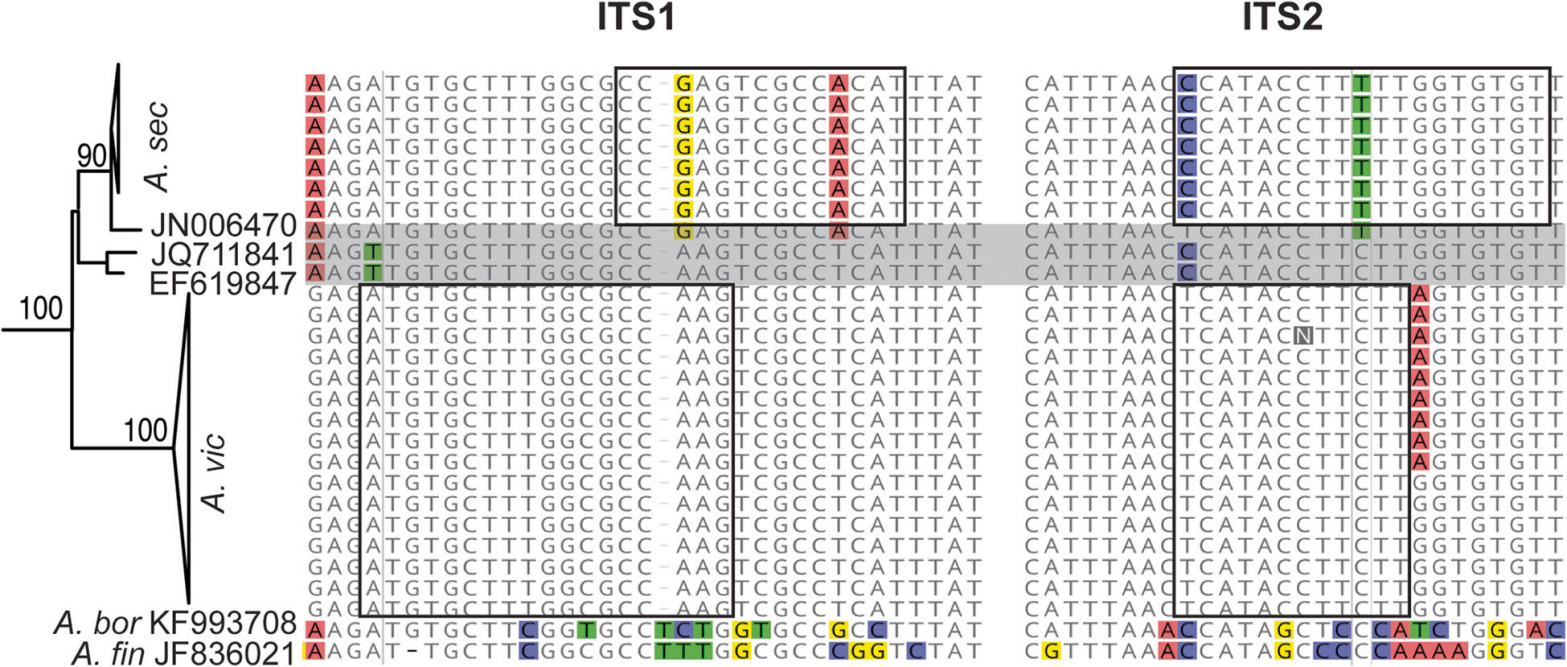
Close up of clade with Arc*haeorhizomyces secundus* (*A. sec*) and *Archaeorhizomyces victor* (*A. vic*) and three closely related environmental sequences ladled with their GenBank accession number. Diagnostic sequences that distinguish the two novel species from each other and from the two described species *Archaeorhizomyces borealis* (*A. bor*) and *Archaeorhizomyces finlayi* (*A. fin*) are highlighted with boxes in a partial alignment of the ITS1 and ITS2 regions. The three closely related environmental sequences are shaded in grey. Alignment start at 1.763 in KF993708 (*A. bor*) and 43 in JF836021 (*A. fin*) for ITS1 region and at 2.035 in KF993708 (*A. bor*) and at 262 in JF836021 (*A. fin*) for ITS2

Based on combined phylogenetic and ecological evidence, we propose two novel species, *Archaeorhizomyces secundus nom. seq.* for SH_7 and *Archaeorhizomyces victor nom. seq.* for SH_8. Names are appended with *nom. seq.* to indicate that the names are based on a sequence in the absence of acceptable type material (Lücking and Hawksworth 2018). We also provide short diagnostic sequences in the ITS1 and ITS2 region to distinguish the two species from each other and from the two described species in the class (Fig. 3).

## TAXONOMY

***Archaeorhizomyces secundus*** *nom. seq.* Kluting, M. Ryberg & Rosling *sp. nov.*

MycoBank MB826774

*Etymology*: At Ivantjärnsheden field station, the species is outnumbered for colonization of the organic soil horizon by its closest known sister taxon *Archaeorhizomycetes victor*.

*Diagnosis*: Separated from other known species in the genus by ribosomal sequences possessing the following distinctive characters in ITS1: CCGAGTCGCCACAT, at position homologous to bases 103–116 in ASV_4; and in ITS2: CCATACCTTTTTGGTGTGT, at position homologous to bases 317–335 in ASV_4, (Fig. 3).

*Type*: rDNA sequence ASV_4 based on sequence reads from Ivantjärnsheden field station, Jädraås, SWEDEN, Uppsala, October-2013, **UDB0779127** in UNITE.

*Description*: Detected by rDNA amplicon sequencing in DNA extracts from soil and from roots, often from ectomycorrhizal roots of *Pinus sylvestris* but also from roots of *Calluna vulgaris*. The species is frequently detected at Ivantjärnsheden field station where it has a patchy distribution across in samples. Found in all studied Podzol soil horizons, it is outnumbered for colonization of the organic soil horizon by its closest known sister taxon *Archaeorhizomycetes victor.*

*Ecology*: Found mostly in pine forests but also in other coniferous forests often with ericaceous understory. Broad climatic distribution including temperate, boreal and alpine climate. Found in both organic and mineral soil horizons but is relatively less abundant in upper organic soil layer compared to *Archaeorhizomyces victor*.

*Distribution*: Austria, Finland, Norway, Sweden, United Kingdom, (Available metadata from publications in Supplementary datafile 7).

*Notes*: Additional sequences, AB560514, DQ309209, FM992980, HM069470, JN032483, KX289979 (Alignment in Supplementary datafile 8).

***Archaeorhizomyces victor*** *nom. seq.* Kluting, M. Ryberg & Rosling sp. nov**.**

MycoBank MB827624

*Etymology*: This species appear to win in competition with its closest known sister taxon for colonization of organic soil at Ivantjärnsheden field station.

*Diagnosis*: Distinct from other known species in the genus by ribosomal sequences possessing the following distinctive characters in ITS1: ATGTGCTTTGGCGCCAAG at position homologous to bases 93–110 in ASV_3; and in ITS2: TCATACCTTCTT at position homologous to 323–333 in ASV_3, (Fig. 3).

*Type*: rDNA sequence ASV_3 based on sequence reads from Ivantjärnsheden field station, Jädraås, SWEDEN, Uppsala, October-2013, **UDB0779126** in UNITE.

*Description*: Detected by rDNA amplicon sequencing in DNA extracts from soil and from roots, often from ectomycorrhizal roots of *Pinus sylvestris* but also from *Calluna vulgaris*. The species is frequently detected at Ivantjärnsheden field station where it is consistently found in both organic and mineral soil horizons, outnumbers closest known sister taxon *Archaeorhizomyces secundus* in upper organic soil layer.

*Ecology*: Most frequently found in coniferous forests but also in deciduous forest. Broad climatic distribution including temperate, boreal and alpine climate.

*Distribution*: Austria, Finland, Germany, The Netherlands, Sweden, United Kingdom (Available metadata from publications in Supplementary datafile 7).

*Notes*: Additional sequences, AB560521, DQ309123, HM069408, HQ873359, JF300381, JN006467, JN006468, JN032485 (Alignment in Supplementary datafile 8).

## DISCUSSION

The class *Archaeorhizomycetes* is diverse, ubiquitous, and often highly abundant in environmental DNA samples from around the world (Rosling et al. 2011). With only two formally named and thus recognized species, very little is known about the ecology, morphology, and life styles of the species within *Archaeorhizomycetes* (Menkis et al. 2014; Rosling et al. 2011). Species within the class are likely to have different ecological roles and occupy different niches in soil horizons (Rosling et al. 2013), but intra-taxonomic boundaries have only begun to be characterized within this lineage. The goal of this study was to investigate the diversity of *Archaeorhizomycetes* at Ivantjärnsheden field station, a well-studied site from which occurrences of OTUs representing species of *Archaeorhizomyces* have been repeatedly documented (Fransson and Rosling 2014; Lindahl et al. 2007). We have demonstrated the notable difficulty associated with obtaining pure cultures of *Archaeorhizomycetes*, even from such site with known high richness and abundance based on amplicon sequencing. This prevents us from collecting traditional sources of evidence for species descriptions, such as morphological characterization, and we have instead delineated two novel species of *Archaeorhizomycetes* using alternative lines of evidence inferred from environmental sequence data.

By combining phylogenetic and ecological evidence, we show that these two species are different from each other and from previously described species. We propose the names *A. secundus nom. seq.* and *A. victor nom. seq.* for them, and have appended the names with *nom. seq.* to represent their status, in line with the recommendations of Lücking and Hawksworth (2018). To avoid future confusion, we plea that these names are continued to be used if and when material becomes available to give them valid names in accordance with ICNafp. It is important to remember the function of a name: as a tool to label and communicate a defined object or concept. We hope that by applying and using these names, we will be able to accumulate knowledge about these species faster, and potentially expediate discovery of reprecentative living specimens.

## CONCLUSION

Diversity estimates indicate that the majority of fungal species remain known only from sequences detected in environmental samples. We demonstrate that environmental sequences and their accompanying metadata can be used as phylogenetic and environmental evidence to build and test species hypothesis. In the absences of physical specimens, environmental sequence data may facilitate species discovery in lineages of abundantly and frequently observed, yet largely unknown, taxa such as the *Archaeorhizomycetes*.

## Supplementary information

**Additional file 1.** Supplementary material

## Data Availability

Sequences generated to screen new cultures are available on GenBank, with accession numbers MH843963–MH844060, (PopSet: 1472857015). Raw reads for both the “phylogenetic” and “ecological” datasets are available in ENA under the accessions ERS3508481- ERS3508483 and ERS4600640-ERS4600675. The complete “phylogenetic” dataset with 272 fungal ASVs is available in UNITE (accession numbers UDB0779092- UDB0779901) and the GBIF repository, 10.15468/8zymuf. Sequence alignments and phylogenetic trees are available in the TreeBASE repository, http://purl.org/phylo/treebase/phylows/study/TB2:S22994. The 1,664 itASVs in the “ecological” dataset are published in GenBank with accession numbers MT926458-MT928121. Additional data supporting the conclusions of this article are included as supplementary datafiles 1-8 are available in our published OSF repository at: 10.17605/OSF.IO/96DKZ.

## ABBREVIATIONS

ASV: Amplicon sequence variant
itASV: ASV from IonTorrent dataset (“ecological dataset”)
SH: Species hypotheses
