# Supplementary material for: Naming the untouchable – environmental sequences and niche partitioning as taxonomical evidence in fungi
Source: IMA Fungus. 2020 Nov 3;11:23. doi: 10.1186/s43008-020-00045-9 (PMC7607712; doi:10.1186/s43008-020-00045-9)
Supplement: Supplementary file 1 — Additional file 1. Supplementary material [file 43008_2020_45_MOESM1_ESM.pdf]

Supplementary material

Naming the untouchable – environmental sequences and niche partitioning as taxonomical evidence in fungi

Faheema Kalsoom Khan[1,2 **], Kerri Kluting [1 **], Jeanette Tångrot [3], Hector Urbina [1,3], Tea Ammunet[1], Shadi Eshghi Sahraei [1], Martin Rydén [1], Martin Ryberg [2], Anna Rosling [1*]

Section **Page**

Supplementary tables

Table S1. Sample names, soil horizon origin, primer number and barcode 2
Table S2. Number of long reads throughout the quality control and filtering 3
Table S3. Overview of 41 *Archaeorhizomycetes* ASVs in "phylogenetic" dataset 4
Table S4. Overview of *Archaeorhizomycetes* reads in the "ecological" dataset 5
Table S5. Summary statistics for 2-way ANOVA for "ecological" dataset 6
Table S6. Summary statistics for PERMANOVA on SHs 6
Table S7. Niche-specific distribution of SHs, Kruskal-Wallis test 7
Table S8. Frequency and abundance of nine *Archaeorhizomycetes* SHs 7
Table S9. Niche-specific distribution across two orthogonal contrasts, MANOVA test 7

Supplementary figures

Figure S1. Overview of sampled plots at Ivantjärnsheden field station 8
Figure S2. All fungal ASV tree identifying ASVs in *Archaeorhizomycetes* 9
Figure S3. nMDS ordination of total fungal itASV community in ugit102 10
Figure S4. Ranked relative abundance and cumulative abundance of SHs in Fig. 1 11
Figure S5. Proportion (%) of *Archaeorhizomycetes* reads across soil horizons 12
Figure S6. Number of itASVs and SHs across soil horizon 13
Figure S7. Tree corresponding to Fig. 1 including node labels 14
Figure S8. nMDS of relative abundance of 68 taxa across soil horizons 17
Figure S9. Relative abundance of *A. finlayi* and SH_7 vs. SH_8 18
Figure S10. Tree corresponding to Fig. 2 including node labels 19

Supplementary methods

Method S1. Field site, soil sampling and isolation efforts 21
Method S2. Soil DNA extraction 22
Method S3. Generating the "phylogenetic" sequence dataset 22
Method S4. Generating the "ecological" sequence dataset 23
Method S5. Delimiting phylogenetic species hypotheses 25
Method S6. Placement of SHs with published sequences 27

Supplementary references 28

Supplementary tables

Table S1. Metabarcoding of the fungal ITS2 region codes for Sample names, soil horizon (Horizon), primer number and barcode used for Ion Torrent sequencing of “ecological” sequence dataset. Right column indicates samples included in PacBio libraries (SWO, SWE and SWB) for the “phylogenetic” sequence dataset.

Plot nr. /cont.	Horizon	Primer nr.	Barcode sequence	In PB library
4	O	39	TAACAATCGGCGAT	
4	B	8	TTCCGATAACGAT	SWB
4	E	84	CTTCCATAACGAT	SWE
17	O	15	TCTAGAGGTCGAT	SWO
17	B	90	CTAACCACGGCGAT	
17	E	29	TCGACCACTCGAT	
1F	O	26	TTACAACCTCGAT	SWO
1F	E	18	AGGCAATTGCGAT	
1F	B	4	TACCAAGATCGAT	
2O	O	63	CCTTAGAGTTCGAT	
2O	E	68	TCAAGAAGTTCGAT	SWE
2O	B	70	CCTACTGGTCGAT	
3I	O	34	TCGCATCGTTCGAT	SWO
3I	E	58	TCCTAGAACACGAT	SWE
3I	B	12	TAGGTGGTTCGAT	
11	O	67	TTCCTACCAGTCGAT	SWO
11	E	27	AACCATCCGCGAT	SWE
11	B	79	CCTGGTTGTCGAT	
12	O	54	CCGGAGAATCGCGAT	
12	E	44	TTGGAGGCCAGCGAT	
12	B	20	CAGATCCATCGAT	
13	O	81	CCTGCCATTCGCGAT	SWO
13	E	19	TTAGTCGGACGAT	SWE
13	B	77	CGAAGCGATTCGAT	SWB
16	O	51	TTGAGCCTATTCGAT	
16	E	55	TCCACCTCCTCGAT	SWE
16	B	32	TCTTACACACGAT	
21	O	37	CTTGAGAATGTCGAT	
21	E	89	TCCTGAATCTCGAT	
21	B	35	TAAGCCATTGTCGAT	
23	O	69	TTCAATTGGCGAT	
23	E	48	TTCTAAGAGACGAT	SWE
23	B	43	CTTGACACCGCGAT	SWB
24	O	92	CTAGGAACCGCGAT	
24	E	86	CTTGGTTATTCGAT	SWE
24	B	62	TTCCTGCTTCACGAT	
ugit.13	Neg. cont.	13	TCTAACGGACGAT	
ugit.14	Pos. cont.	14	TTGGAGTGTCGAT	

Table S2. Number of long reads and amplicon sequence variants (ASVs) from initial PacBio CCS reads throughout the quality control and filtering steps used to generate a sequence dataset representing the *Archaeorhizomycetes* (Arch.) diversity at the study site. Numbers are presented for the three sequenced libraries (SWO, SWE, SWB) separate and combined (Across libraries). ASVs taxonomically assigned to *Archaeorhizomycetes* were aligned and manually checked for chimers, this identified one chimeric ASV (ASV_135) that was removed. In the end, the *Archaeorhizomycetes* "phylogenetic" sequence dataset (Included Arch. ASVs) represented 52,274 reads across three libraries.

**Steps**	**SWO**	**SWE**	**SWB**	**Across libraries**
PacBio CCS	67,159	69,083	61,914	198,156
ASVs with ITS2 region	208	239	178	276
ASVs incl. All fungal ASVs	207	236	178	273
ASVs assigned to Arch.	33	35	31	42
Chimeric ASV_135	1	1	1	1
Included Arch. ASVs	32	34	30	41
Reads represented by Arch. ASVs	21,324	13,302	17,648	52,274
Rel. ab. Arch.	32%	19%	29%	26%

Table S3. Summary of 41 *Archaeorhizomycetes* ASVs in phylogenetic dataset

	Total	SWO	SWE	SWB		
Fungi ASVs with ITS	112485	35526	38767	38192		
Tot Arch. seq	**52274**	21324	13302	17648		
Rel abund Arch.	**46%**	**60%**	**34%**	**46%**		
nr of Arch. ASVs	**42**	33	35	31		
**ASV ID**	**Total**	**SWO**	**SWE**	**SWB**	**SH**	**UNITE acc.nr**
ASV_1	14049	262	395	13392	SH_9 A.fin	UDB0779122
ASV_3	9472	5630	3468	374	SH 8	UDB0779126
ASV_4	7837	5862	1089	886	SH 7	UDB0779127
ASV_5	4916	1503	2348	1065	SH 1	UDB0779128
ASV_7	2935	2093	832	10	SH 2	UDB0779130
ASV_8	1971	1922	49		SH 2	UDB0779131
ASV_12	1797	1326	353	118	SH 8	UDB0779135
ASV_19	1111	1078	30	3	SH 1	UDB0779140
ASV_23	811	6	805		SH 1	UDB0779143
ASV_24	785	20	765		SH 2	UDB0779144
ASV_29	681	80	601		SH 2	UDB0779149
ASV_31	604	183	29	392	SH 3	UDB0779151
ASV_35	533	1	532		SH 2	UDB0779155
ASV_37	507	500	4	3	SH 8	UDB0779157
ASV_38	505	9	495	1	SH 2	UDB0779158
ASV_48	371	4	159	208	SH 2	UDB0779168
ASV_59	278		1	277	SH_9 A.fin	UDB0779179
ASV_63	266	3	227	36	SH 1	UDB0779183
ASV_66	261	241	11	9	SH 8	UDB0779111
ASV_68	251	2	231	18	SH 1	UDB0779186
ASV_73	241	4	234	3	SH 5	UDB0779191
ASV_82	219	2	106	111	SH 2	UDB0779200
ASV_84	213	138	32	43	SH 8	UDB0779202
ASV_86	202	20	179	3	SH 5	UDB0779204
ASV_88	193	140	30	23	SH 8	UDB0779206
ASV_90	184	5	32	147	SH 6	UDB0779208
ASV_104	154	79		75	SH 3	UDB0779221
ASV_106	149	149			SH 3	UDB0779223
ASV_120	121		121		SH 5	UDB0779237
ASV_122	118			118	SH_9 A.fin	UDB0779239
ASV_134	100			100	SH_9 A.fin	UDB0779251
ASV_136	98			98	SH 3	UDB0779113
ASV_155	76			76	SH 3	UDB0779269
ASV_176	57	9	48		SH 5	UDB0779287
ASV_181	53	2	51		SH 8	UDB0779292
ASV_210	36	28	4	4	SH 8	UDB0779318
ASV_220	32		32		SH 6	UDB0779328
ASV_230	25		1	24	SH 3	UDB0779338
ASV_238	21	2	3	16	SH 5	UDB0779346
ASV_239	21	21			SH 4	UDB0779901
ASV_240	20		5	15	SH 3	UDB0779347
Removed sequence						
ASV_135 Chimer	100	94	2	4		

Table S4. Overview of *Archaeorhizomycetes* reads in the "ecological" dataset

Plot	Horizon	Fungal reads	Rel. ab. Arch. (%)	Arch. reads	Arch. itASVs	Arch. SHs	Arch. SHs without long reads	
							Rel. ab. (%)	Nr. of SHs
1	B	22,967	29	6,613	30	12	0.3	5
1	E	28,885	34	9,680	20	7	0.0	0
1 *	O	19,882	32	6,283	23	9	0.4	4
2	B	1,175	10	113	19	11	0.5	3
2 *	E	22,255	63	13,978	24	9	0.0	2
2	O	22,950	46	10,567	25	10	1.2	2
3	B	20,046	30	5,984	21	10	0.0	4
3 *	E	21,285	45	9,574	21	6	0.0	0
3 *	O	20,297	39	7,941	19	10	0.1	2
4 *	B	24,477	41	10,132	38	19	0.4	11
4 *	E	22,234	31	6,797	27	15	0.2	9
4	O	17,508	34	6,040	16	8	0.1	3
11	B	5,673	21	1,202	17	12	0.1	5
11 *	E	17,747	31	5,465	12	4	0.0	0
11 *	O	9,569	28	2,643	17	7	0.0	0
12	B	1,141	14	165	18	14	1.2	9
12	E	15,263	0	3	3	3	0.0	0
12	O	16,290	4	684	20	16	0.2	13
13 *	B	23,884	59	14,128	26	10	0.1	2
13 *	E	22,283	72	15,976	17	8	0.0	0
13 *	O	23,818	44	10,508	19	10	0.6	2
16	B	12,695	27	3,384	36	24	2.7	16
16 *	E	20,181	43	8,672	13	7	0.0	1
16	O	9,845	29	2,903	16	8	0.1	2
17	B	24,957	55	13,760	57	31	31.3	22
17	E	20,622	37	7,722	38	21	0.4	12
17 *	O	20,329	38	7,708	21	10	0.4	3
21	B	828	6	53	16	10	1.0	6
21	E	11,915	30	3,545	17	8	0.0	2
21	O	8,682	52	4,531	20	11	0.1	5
23 *	B	9,143	10	874	21	13	0.2	8
23 *	E	22,783	43	9,888	22	9	0.2	3
23	O	18,786	42	7,804	13	5	4.2	1
24	B	22,155	53	11,791	49	32	42.0	26
24 *	E	15,570	16	2,563	13	7	0.0	3
24	O	21,056	18	3,693	13	6	0.0	3
	Average	17,199	33	6,483	22	12	2	5

Plot – Plot number, * indicates that the sample was included in "phylogenetic" dataset
Horizon – organic (O), mineral elluvial (E) and mineral illuvial (B)
Fungal reads – number of fungal reads
Rel. ab. Arch. (%) – relative abundance of *Archaeorhizomycetes*
Arch. reads – number of *Archaeorhizomycetes* reads
Arch. itASVs – number of itASVs assigned to *Archaeorhizomycetes*
Arch. SHs – number of SH assigned to *Archaeorhizomycetes*
Arch. SHs without long reads –*Archaeorhizomycetes* SHs not represented in "phylogenetic" dataset. In two columns: **Rel. Ab. (%)** is the relative abundance and **Nr. of SHs** is the number of SH detected only in the "ecological" dataset.

Table S5. Summary statistics for 2-way ANOVAs on number of fungal reads, number of *Archaeorhizomycetes* itASVs and SHs as well as relative abundance of *Archaeorhizomycetes* reads out of all fungal reads across Treatment and Horizon (sequentially added). Significant effects are indicated by *.

Variable		df	Sum Sq.	Mean Sq.	*F*-value	Pr(>F)
**Nr. of fungal reads**	Treatment	3	1.681e+08	56021477	1.149	0.346
	Horizon	2	2.162e+08	108075315	2.216	0.127
	Residual	30	1.463e+09	48776781		
**Nr. of Arch. itASVs**	Treatment	3	138.1	46.0	0.485	0.695
	Horizon	2	848.4	424.2	4.472	0.020 * [1]
	Residual	30	2845.8	94.9		
**Nr. of Arch. SHs**	Treatment	3	93.8	31.26	0.916	0.445
	Horizon	2	514.7	257.33	7.542	0.002 ** [2]
	Residual	30	1023.6	34.12		
**% reads in Arch.**	Treatment	3	1.747e-06	5.823e-07	0.849	0.478
	Horizon	2	3.217e-06	1.609e-06	2.345	0.113
	Residual	30	2.058e-05	6.860e-07		

1. Tukey-adjusted pairwise tests revealed that the number of *Archaeorhizomycetes* itASVs was different in B-horizon compared to the other horizons.
2. Tukey-adjusted pairwise tests revealed that the number of *Archaeorhizomycetes* SHs was different in B-horizon compared to the other horizons.

Table S6. PERMANOVA testing for variables that significantly affect the relative abundance of nine SHs. Model selection started with a full model with all variables (Horizon, Treatment, Plot) and their interactions. Variables were reduced by omitting the highest order non-significant term at each step.

	df	χ^2	*F*-value	Pr(>F)
**Horizon**	2	0.43471	**4.6070**	<0.001 ***
**Plot**	1	0.1896	4.0190	<0.001 ***
**Treatment**	3	0.5680	4.0130	<0.001 ***
**Plot*Treatment**	3	0.2880	2.0344	<0.005 **
**Residual**	26	1.22665		

Table S7.
Kruskal-Wallis tests of niche-specific distribution for nine SHs (supported by long read data) based on the relative read abundance in the “ecological” dataset as the response variable and horizon as the explanatory variable. The *p*-values were corrected for multiple comparisons using the Benjamini & Hochberg method. Corrected *p*-values below 0.1 denoted by •.

**Kruskal-Wallis test**	χ^2	**df**	**Corrected P-value**
SH_1	2.1613106	2	0.6109
SH_2	1.6028322	2	0.6730
SH_3	3.9627832	2	0.4137
SH_4	0.8558524	2	0.7334
SH_5	1.1127558	2	0.7334
SH_6	3.0828607	2	0.4817
SH_7	0.4166110	2	0.8120
SH_8	5.2672673	2	0.3231
SH_9 *A. finlayi*	9.4570478	2	0.0792•

Table S8. Frequency of observation, mean and maximum relative abundance in “ecological” dataset of *Archaeorhizomycetes* SHs supported by long read “phylogenetic” dataset.

**SH**	**Nr of sample**	**Ave rel. abundance**	**Max relative abundance**
SH_1	23	3%	30%
SH_2	27	5%	34%
SH_3	21	1%	24%
SH_4	6	0%	1%
SH_5	34	5%	37%
SH_6	23	1%	6%
SH_7	24	4%	36%
SH_8	36	9%	38%
SH_9 A.fin	29	4%	34%

Table S9. Niche-specific distribution in two orthogonal contrasts defined from the phylogenetic tree in Fig. 1. SH number are listed for the two pairs of sister-SHs. Model results with degrees of freedom (df), sums of squares, mean square sums, *F*-values, R^2-values and Pr(>F)-values are shown, after correcting for multiple comparisons. Corrected probabilities below 0.05 are marked with an asterisk (*) and those below 0.1 with dot (•).

	**df**	**Sum.Sq.**	**Mea.Sq.**	***F*-value**	R^2	**Pr(>F)**
**SH_2 vs. SH_3**	1	0.6380	0.63805	1.7717	0.03878	0.09950•
**SH_7 vs. SH_8**	1	0.6884	0.68842	1.9116	0.04184	0.02985*
**Residual**	42	15.1253	0.36013		0.91937	
**Total**	44	16.4518			1.00000	

Supplementary figures

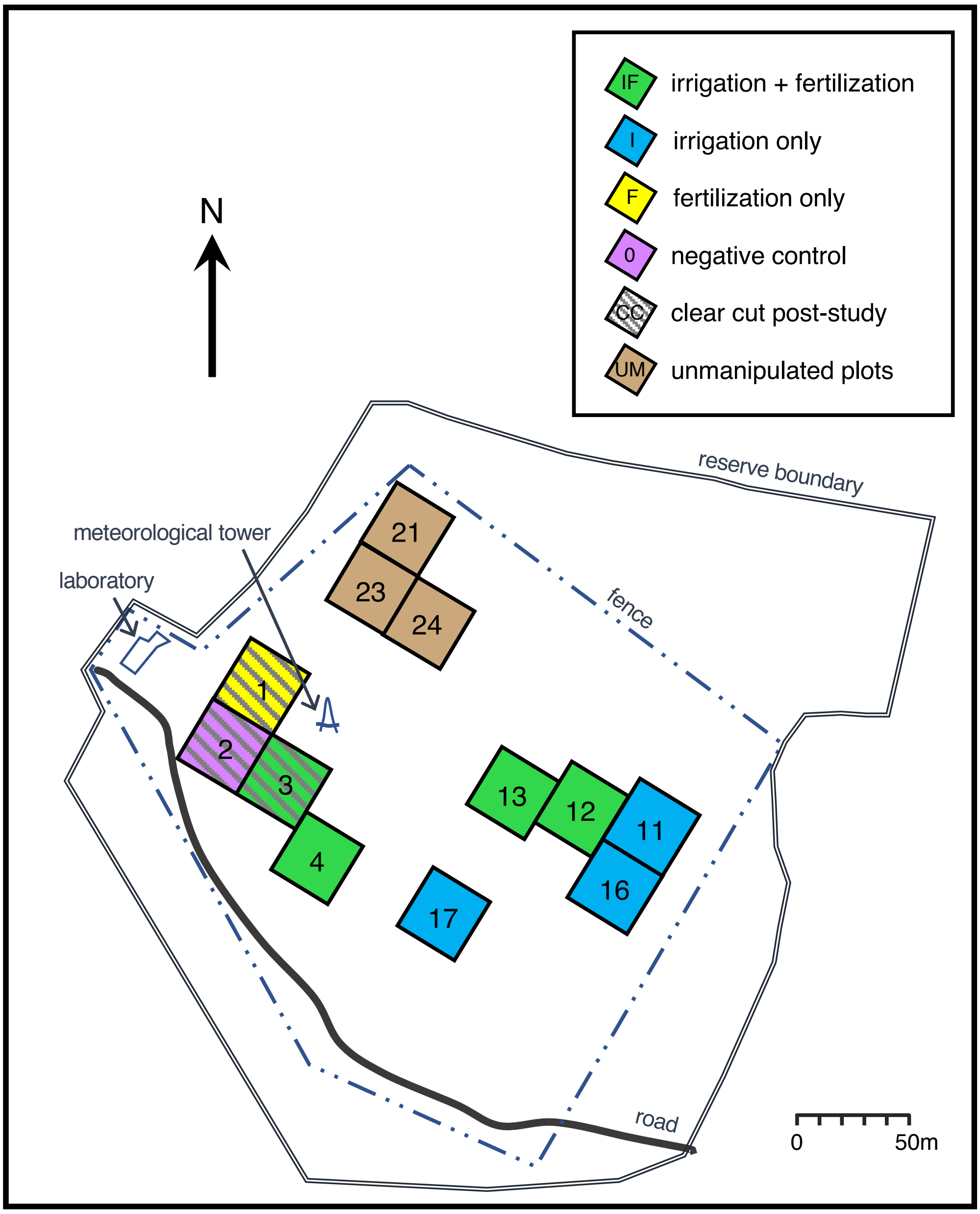

Figure S1
Schematic view of plots sampled at Ivantjärnsheden field station in Jädraås. Redrawn from (Axelsson & Bråkenhielm, 1980). The stand was naturally regenerated after tree-felling in 1957 and thinned before the onset of an experimental field study conducted between 1974 - 1990 (experiment Ih2 (9802)). In the study 30 x 30 m plots received one of three treatments (irrigation and fertilization (IF) in green, irrigation (I) in blue, un-manipulated control (UM) in brown as well as clear cut plots (CC) striped), labeled with plot number. The clear-cut (grey stripes) was part of a second study following the initial experiment and represent one plot from each previous treatment. Forest has since started to regenerated in clear-cut plots.

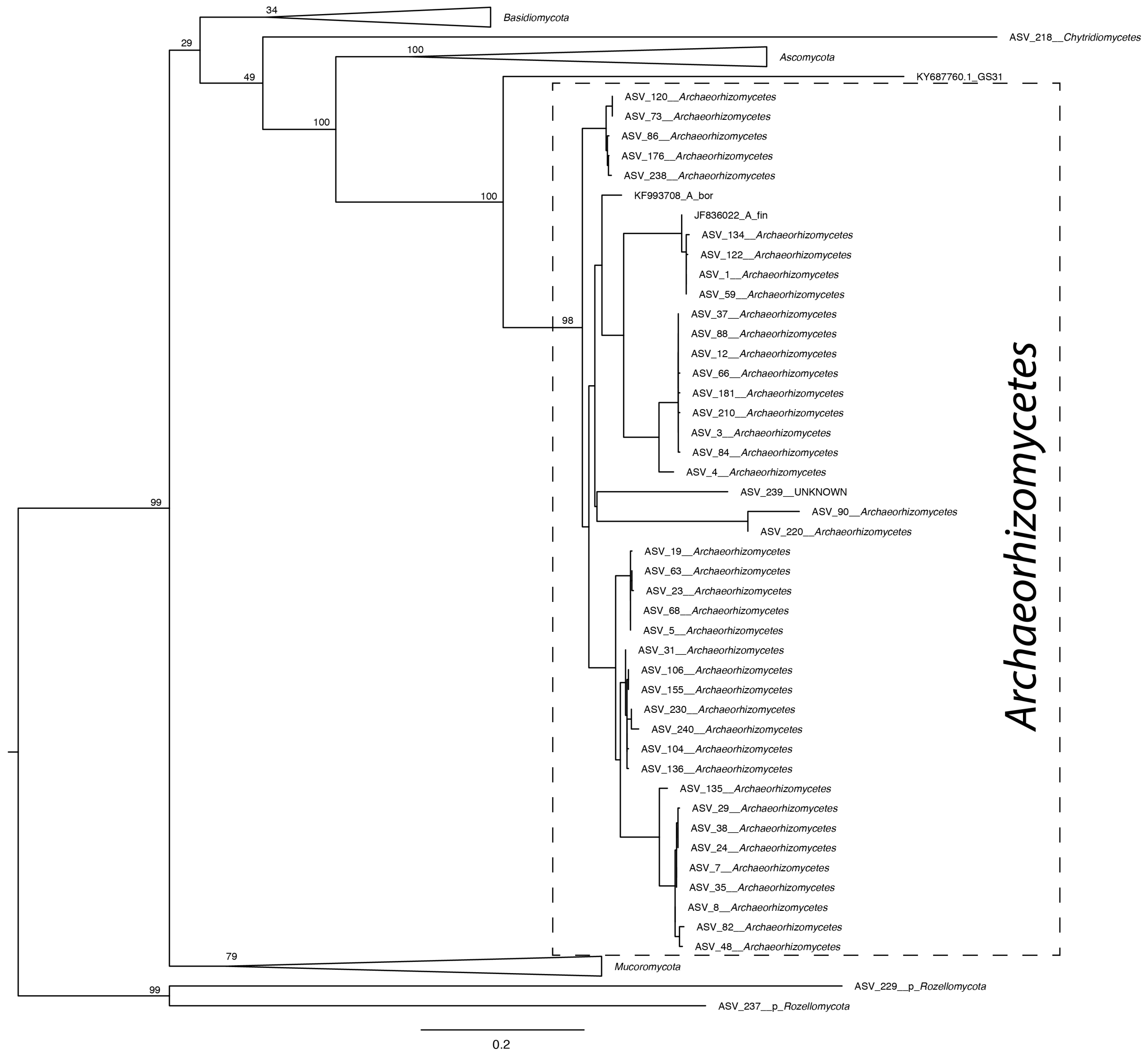

Figure S2

A maximum likelihood tree based on an alignment of the rDNA LSU region of 273 fungal ASVs from the current study as well as reference sequences *A. borealis* (KF993708), *A. finlayi* (JF836022) and the uncultured lineage GS31 (KY687760) which represents a sister class of *Archaeorhizomycetes*. Class level taxonomy based on the SINTAX classifier in USEARCH is amended to the ASV label when assigned. Unclassified sequences are amended with UNKNOWN. Bootstrap support values are calculated from 1000 iterations and shown on the *Archaeorhizomycetes* branch and all deeper branches. Collapsed clades represent *Basidiomycota* (including 106 ASVs), *Ascomycota* outside the *Taphrinomycotina* (111 ASVs) and *Mucoromycota* (11 ASVs). Forty-two ASV LSU regions representing *Archaeorhizomycetes* form a well-supported sister clade to GS31 and are highlighted by a box on the tree.

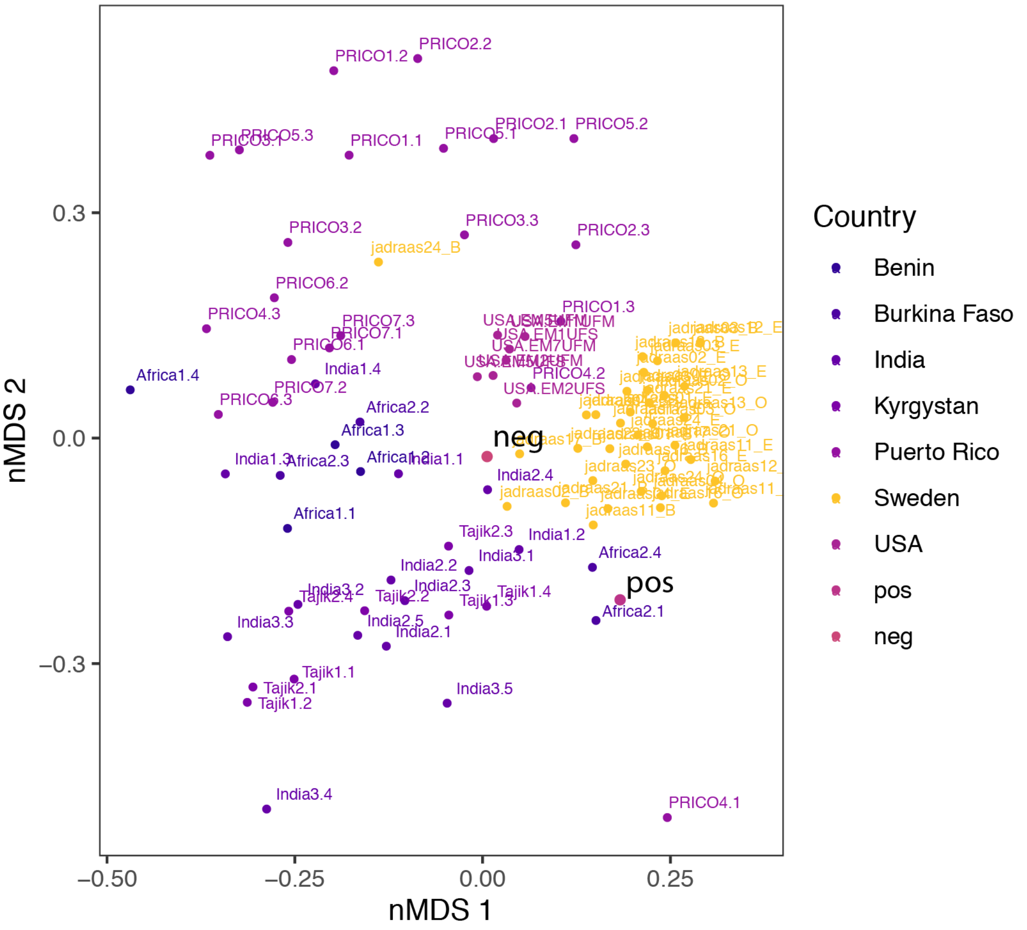

Figure S3

Non-parametric multidimensional scaling (nMDS) ordination displaying communities (fungal ASVs only, positive control sequence (itASV_1) removed; 4,461 ASVs / 1,680,044 sequence reads included) from all 96 samples sequenced on the ugit_102 IonTorrent sequencing run (plot stress = 0.24 based on a maximum of 200 random starts). Prior to ordination, the itASV by sample count matrix was standardized to per-sample relative proportions, and a dissimilarity matrix was calculated using the Bray-Curtis dissimilarity index. Samples in the current study (in yellow) was sequenced on an IonTorrent chip together with samples from several other studies including unpublished samples from Benin, Burkina Faso, India and Kyrgystan, as well as published data from Puerto Rico (Urbina *et al.* 2016) and IN, USA (Rosling *et al*. 2016). Positive (pos) and negative (neg) controlls were also included. Samples generally cluster by study site. However jadraas24_B clusters with samples from Puerto Rico possibly indicating tag-swithing affecting this particular sample.

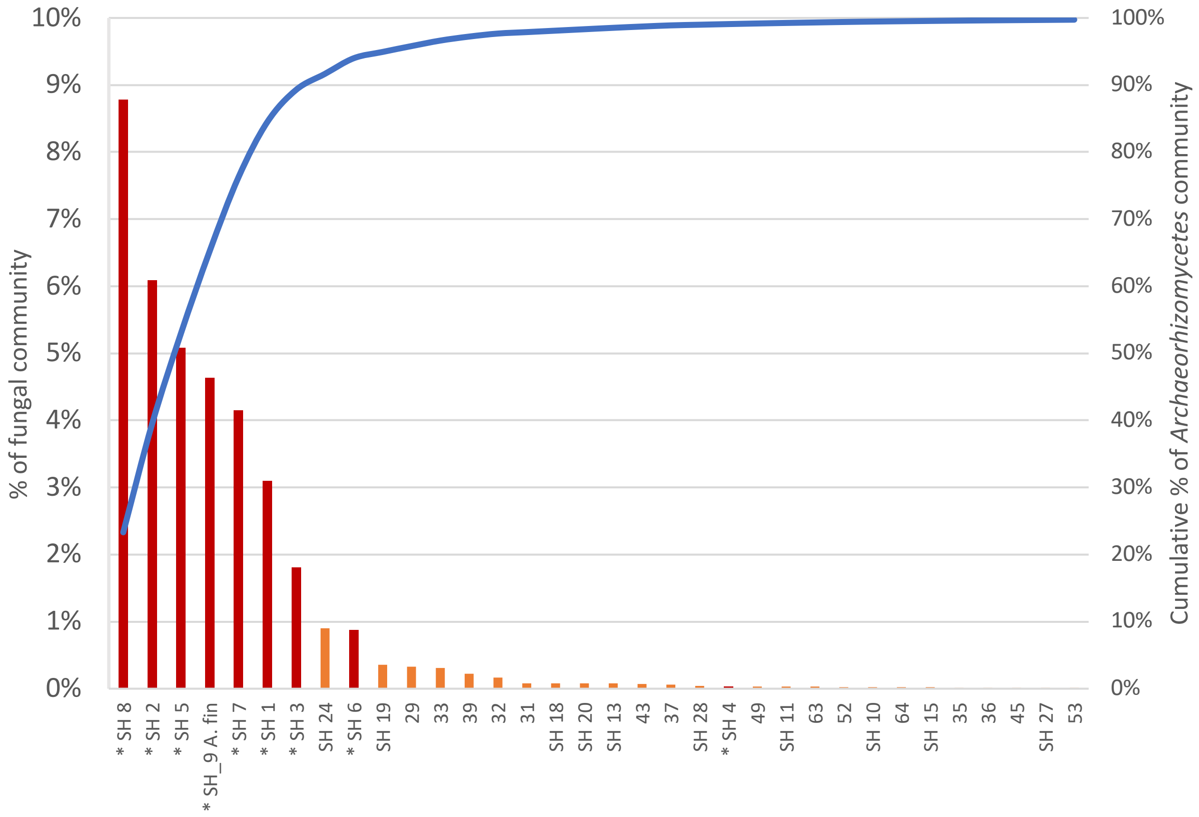

Figure S4
Relative sequence read abundance of the 34 most abundant *Archaeorhiozmycetes* species hypotheses (SH), delimited based on long- and short- environmental amplicon sequences from soil sampled in a south central Swedish pine forest. These represent half of the total number of SHs delimited at the site. Primary axis illustrates percentage of each SH out of the total fungal community based on reads across all 36 samples. SHs highlighted in dark red and indicated with an * before its label are supported by data from both long amplicon data from the "phylogenetic" dataset and short ampicon data from the "ecological" dataset. Secondary axis illustrates cumulative percentage of each SH as a propostion of the total *Archaeorhizomycetes* community at the site, based on sequence reads in the "ecological" dataset.

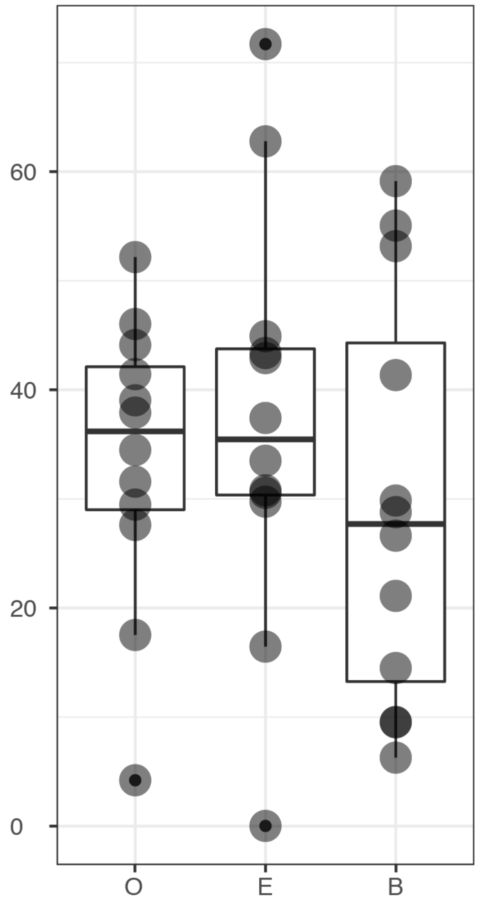

Figure S5
Proportion (%) of reads assigned to class *Archaeorhizomycetes* out of all reads assigned to kingdom Fungi in the "ecological" dataset. Box-and-whiskers plots based on 12 samples from each of the soil horizons, organic soil (O), elluvial mineral soil (E) and illuvial mineral soil (B) with middle line at median, hinges at 25th and 75th percentile and whiskers extending 1.5 * inter-quartile range. Samples are visualized as grey dots and are darker where overlapping and outliers have dark centers.

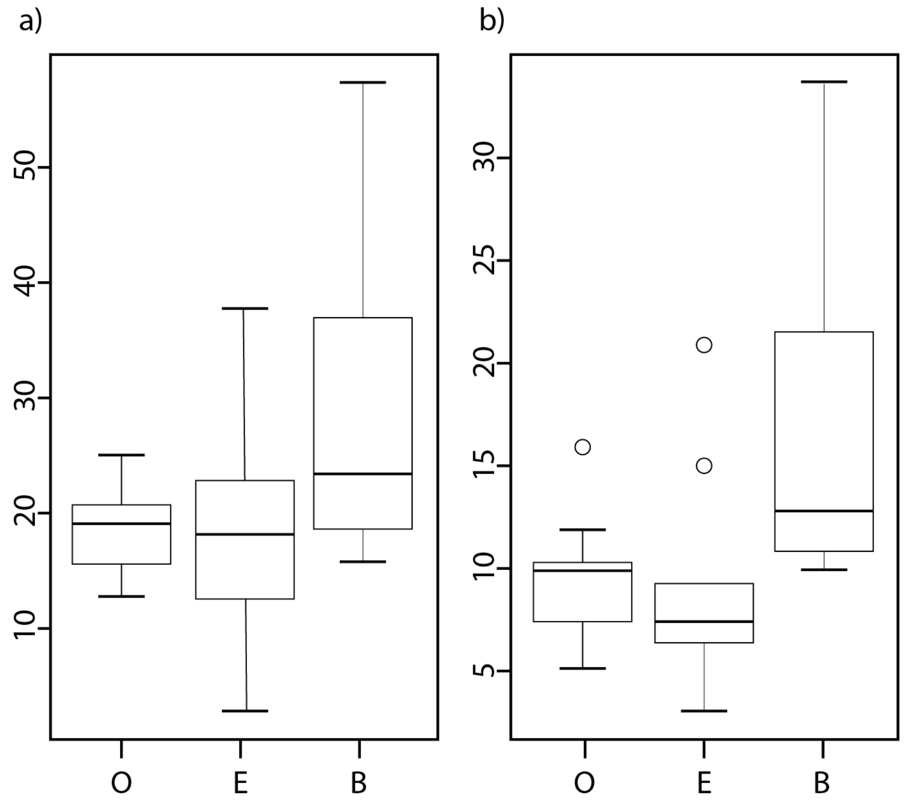

Figure S6
A box-and-whiskers plot depicting the medians, first and third quantile and maximum and minimum of a) number of *Archaeorhizomycetes* itASVs and b) number of *Archaeorhizomycetes* SHs across soil horizons, organic soil (O), elluvial mineral soil (E) and illuvial mineral soil (B). These variables were significantly different based on 2-way ANOVA (Table S5). Outliers are represented as circles.

a)

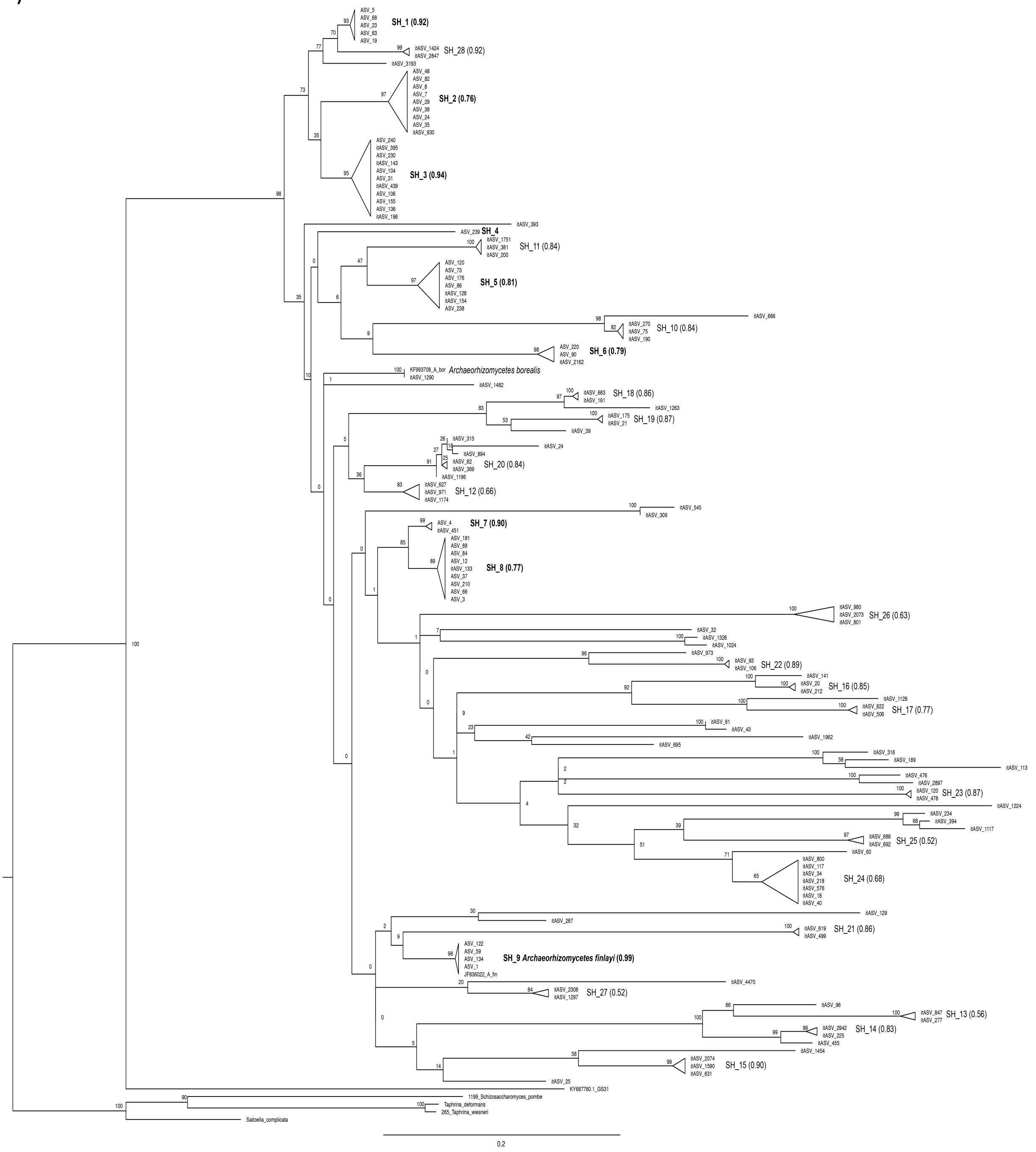

b)

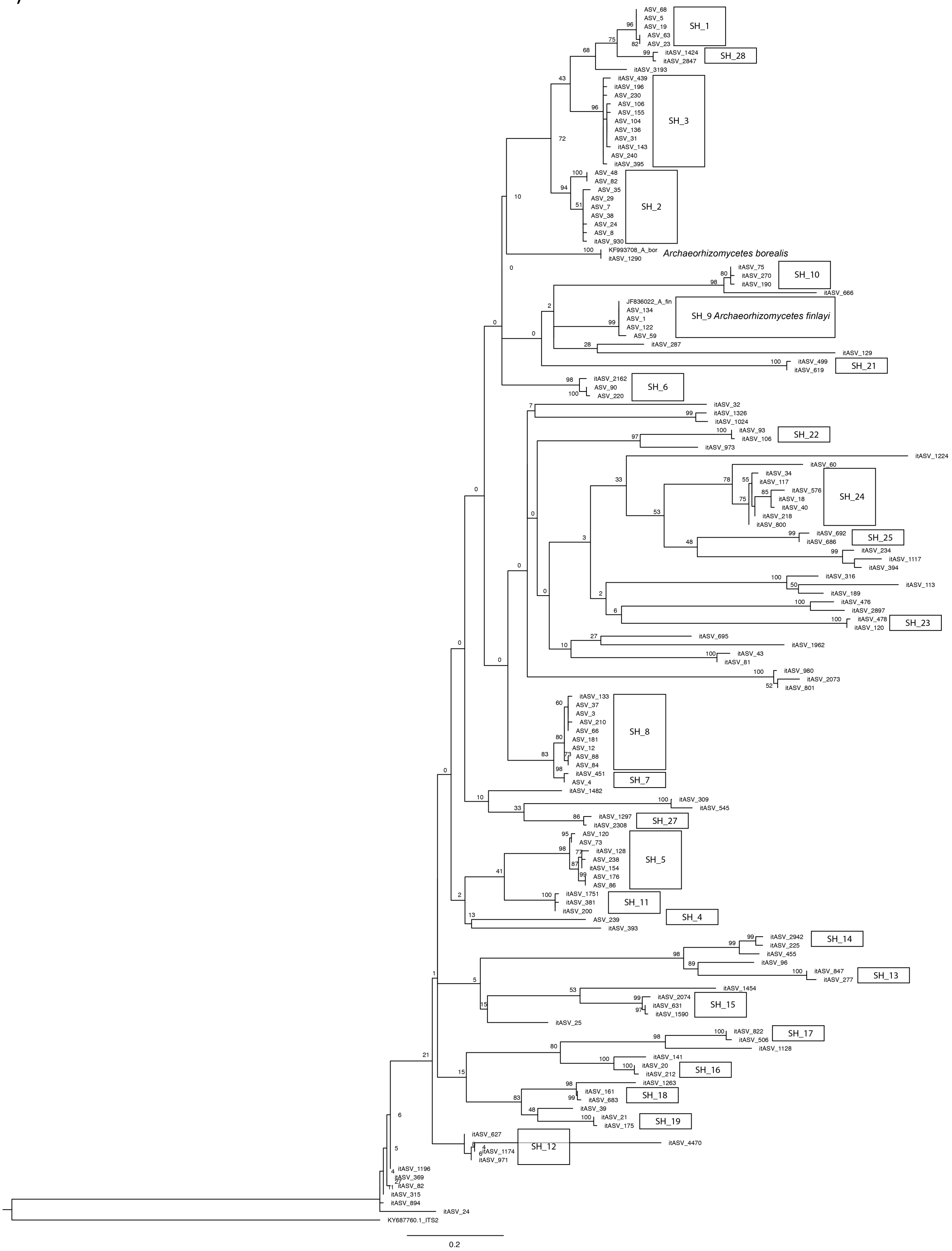
SH_1
SH_28
SH_3
SH_2
Archaeorhizomycetes borealis
SH_10
SH_9 Archaeorhizomycetes finlayi
SH_21
SH_6
SH_22
SH_24
SH_25
SH_23
SH_8
SH_7
SH_27
SH_5
SH_11
SH_4
SH_14
SH_13
SH_15
SH_17
SH_16
SH_18
SH_19
SH_12
0.2

c)

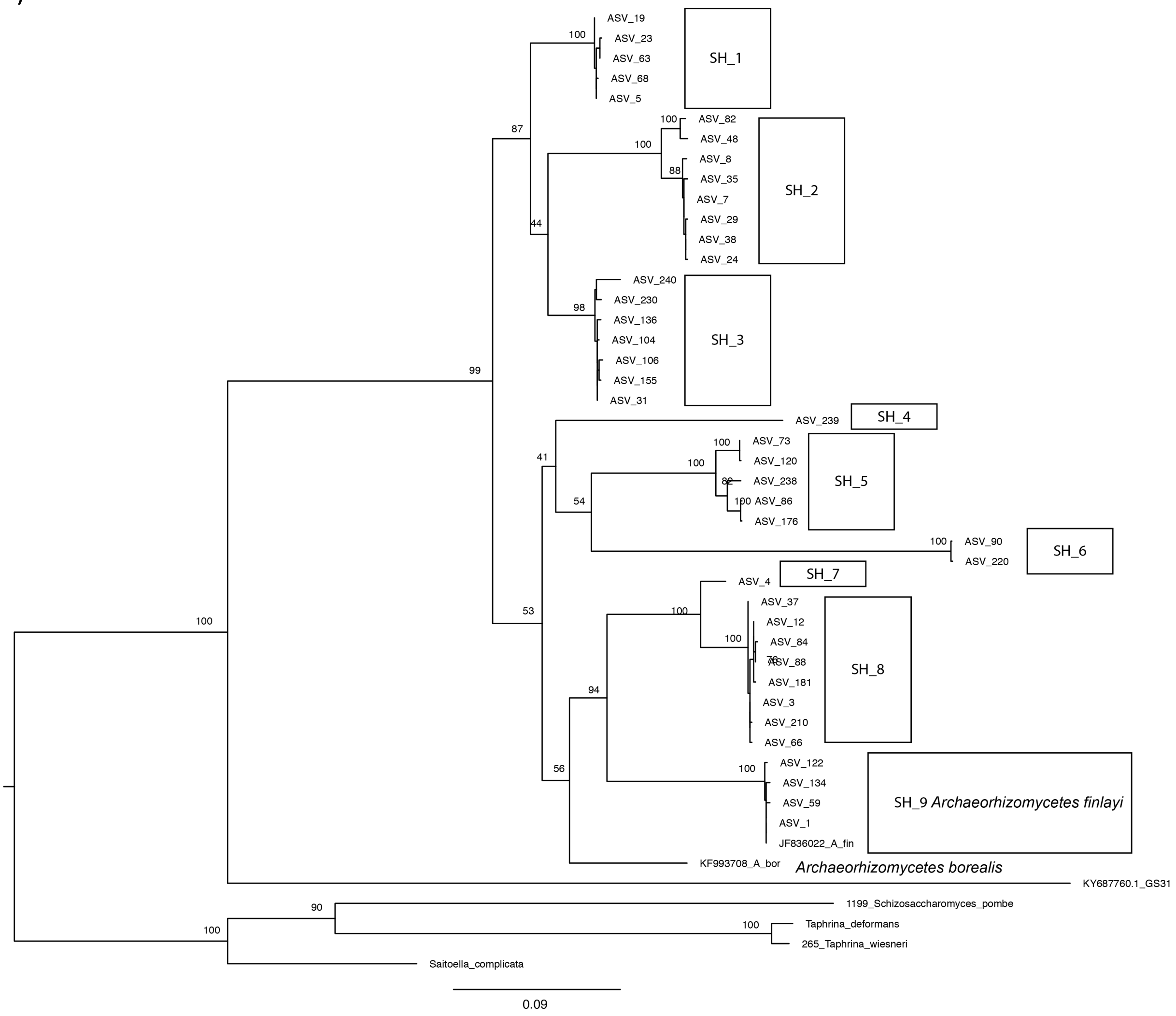

Figure S7

Diversity of *Archaeorhizomycetes* in mid-Sweden Pine forest podzol soil a) illustrated in maximum likelihood tree based on environmental ribosomal long and short amplicon ASVs representing 68 species hypotheses (SHs) including both described species *Archaeorhizomyces borealis* and *Archaeorhizomyces finlayi*. The sister taxa GS31 and four *Taphrinamycotina* species as outgroup. Nodes are cartooned to visualize SHs represented by more than one ASV. b) Maximum likelihood tree based on only the ITS2 region of the same dataset as above with only the sister taxon GS31 as outgroup. Species hypothesis from a) are illustrated with labled boxes next to the included nodes. c) Maximum likelihood tree based on long reads from the same dataset as above. Same outgroup as in a). Species hypothesis from a) are illustrated with labled boxes next to the included nodes. All *Archaeorhizomycetes* ASV from “phylogenetic” and “ecological” dataset are labled at the nodes. Bootstrap support are calculated from 1000 iterations. Tree files are available at https://doi.org/10.17605/OSF.IO/96DKZ

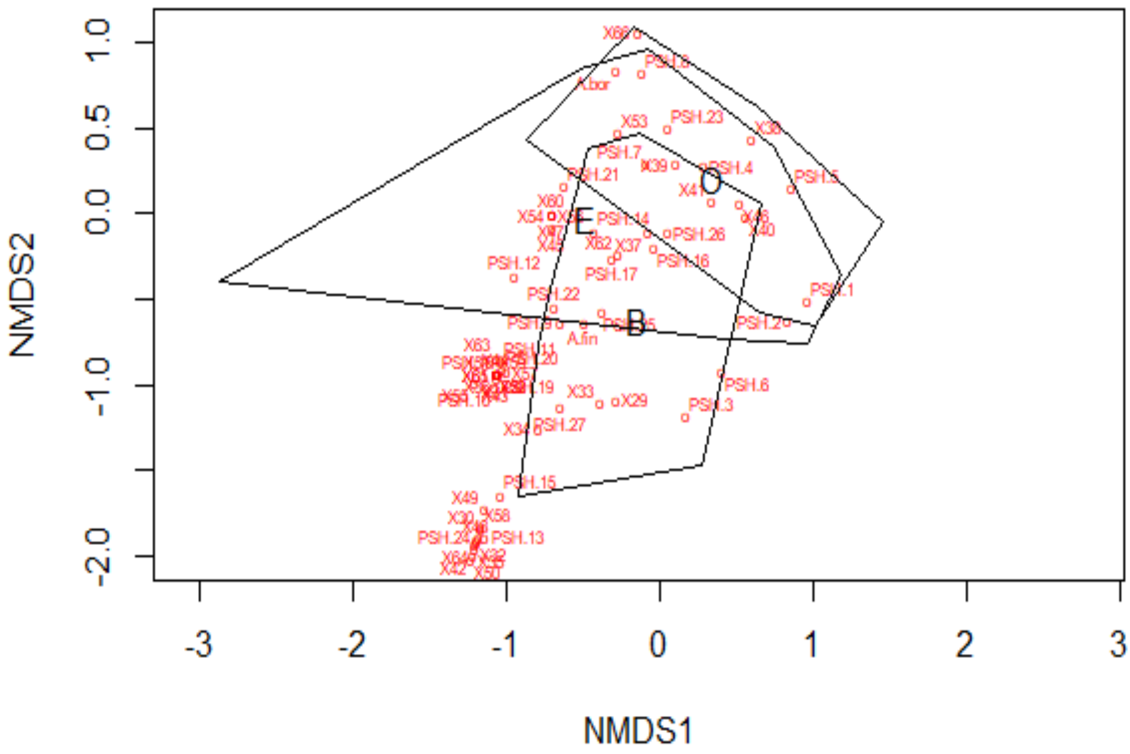

Figure S8

Results from nMDS analysis based on the relative abundance out of sequenced fungal community of 68 delimitated *Archaeorhizomycetes* SHs (red circles labeled SH_number, A.fin, A.bor or Xnumber (for single itASVs)). The soil horizons O (organic), E (elluvial mineral soil) and B (illuvial mineral soil) capturing the measured abundances are marked with their respective convex hulls. Stress value for the nMDS is 0.2058808.

a)

b)

c)

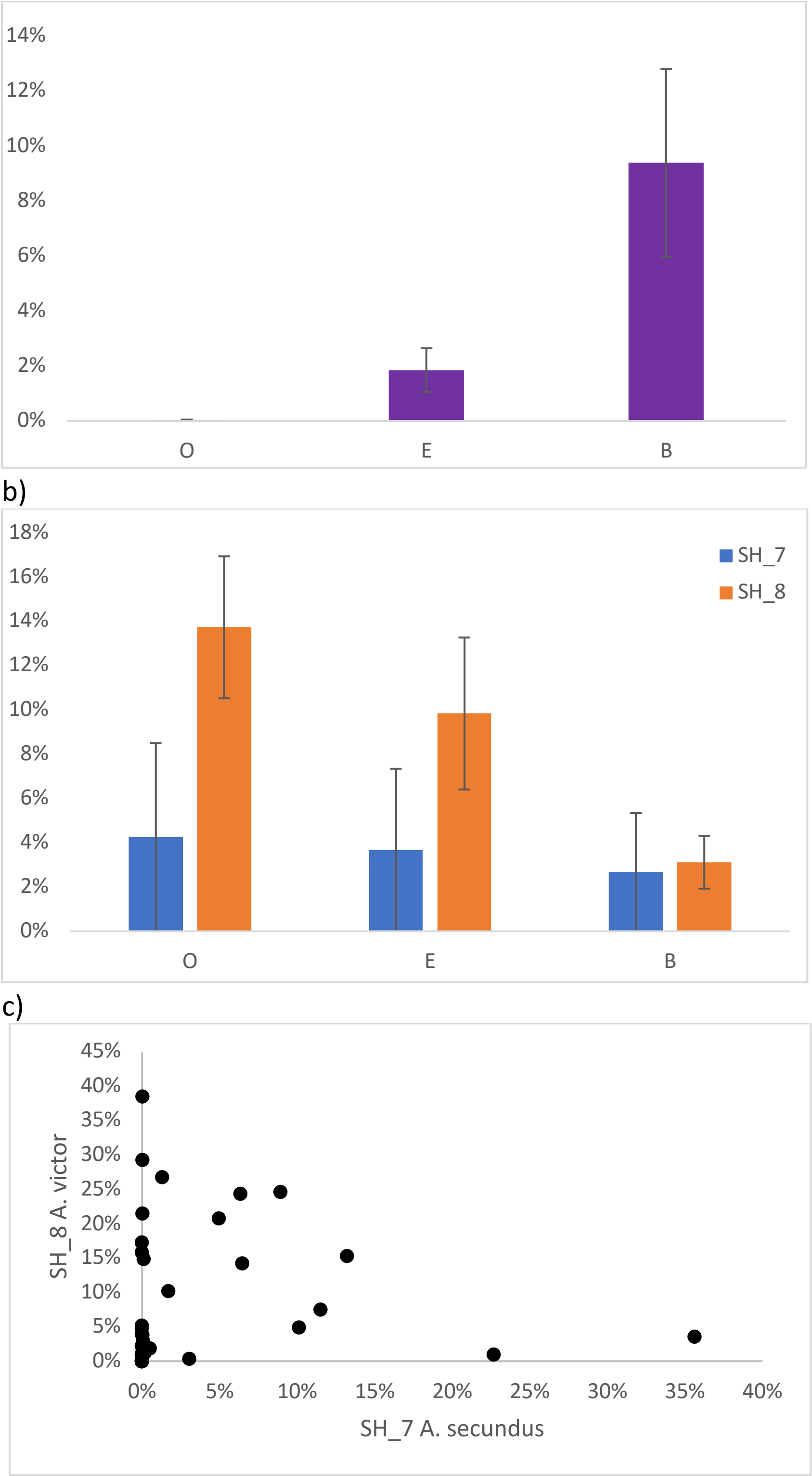

Figure S9

Relative abundance of SH in % out of sequenced fungal community in "ecological dataset". Average for each soil horizons [organic soil (O), elluvial mineral soil (E) and illuvial mineral soil (B)] with SEM in error bars in a) SH_9 *A. finlayi* in purple and b) SH_7 (blue) vs SH_8 (orange). c) relative abundance of the two sister SHs, SH_8 *A. victor* and SH_7 *A. secundus*, across all 36 samples included in the current study.

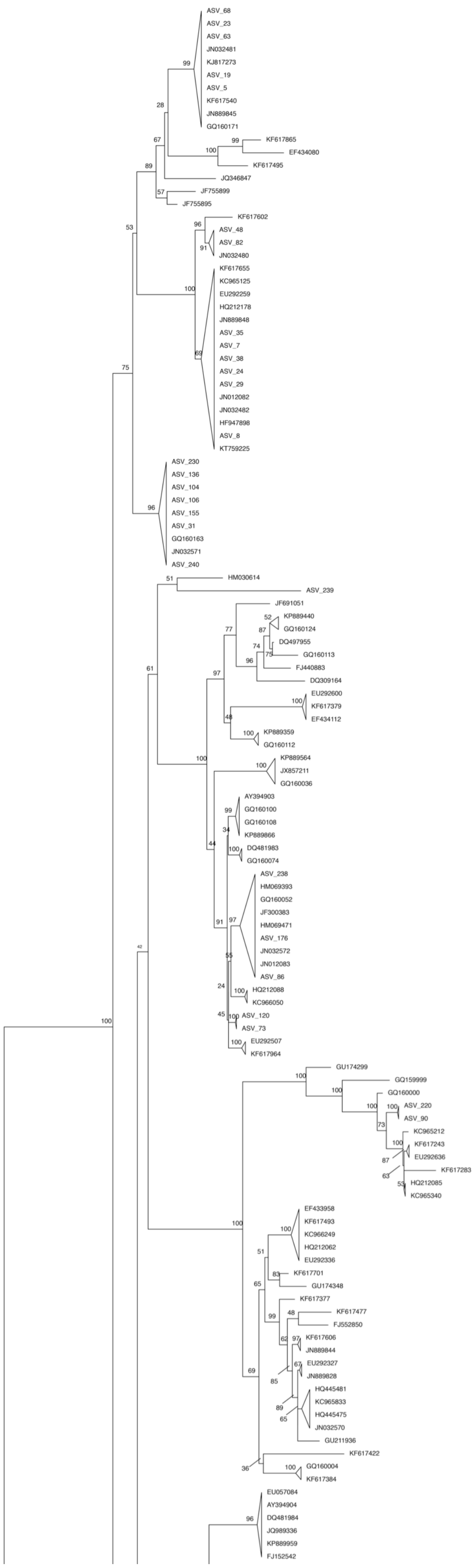
ASV_68
ASV_23
ASV_63
JN032481
KJ817273
ASV_19
ASV_5
KF617540
JN889845
GQ160171
KF617865
EF434080
KF617495
JQ346847
JF755899
JF755895
KF617602
ASV_48
ASV_82
JN032480
KF617655
KC965125
EU292259
HQ212178
JN889848
ASV_35
ASV_7
ASV_38
ASV_24
ASV_29
JN012082
JN032482
HF947898
ASV_8
KT759225
ASV_230
ASV_136
ASV_104
ASV_106
ASV_155
ASV_31
GQ160163
JN032571
ASV_240
HM030614
ASV_239
JF691051
KP889440
GQ160124
DQ497955
GQ160113
FJ440883
DQ309164
EU292600
KF617379
EF434112
KP889359
GQ160112
KP889564
JX857211
GQ160036
AY394903
GQ160100
GQ160108
KP889866
DQ481983
GQ160074
ASV_238
HM069393
GQ160052
JF300383
HM069471
ASV_176
JN032572
JN012083
ASV_86
HQ212088
KC966050
ASV_120
ASV_73
EU292507
KF617964
GU174299
GQ159999
GQ160000
ASV_220
ASV_90
KC965212
KF617243
EU292636
KF617283
HQ212085
KC965340
EF433958
KF617493
KC966249
HQ212062
EU292336
KF617701
GU174348
KF617377
KF617477
FJ552850
KF617606
JN889844
EU292327
JN889828
HQ445481
KC965833
HQ445475
JN032570
GU211936
KF617422
GQ160004
KF617384
EU057084
AY394904
DQ481984
JQ989336
KP889959
FJ152542

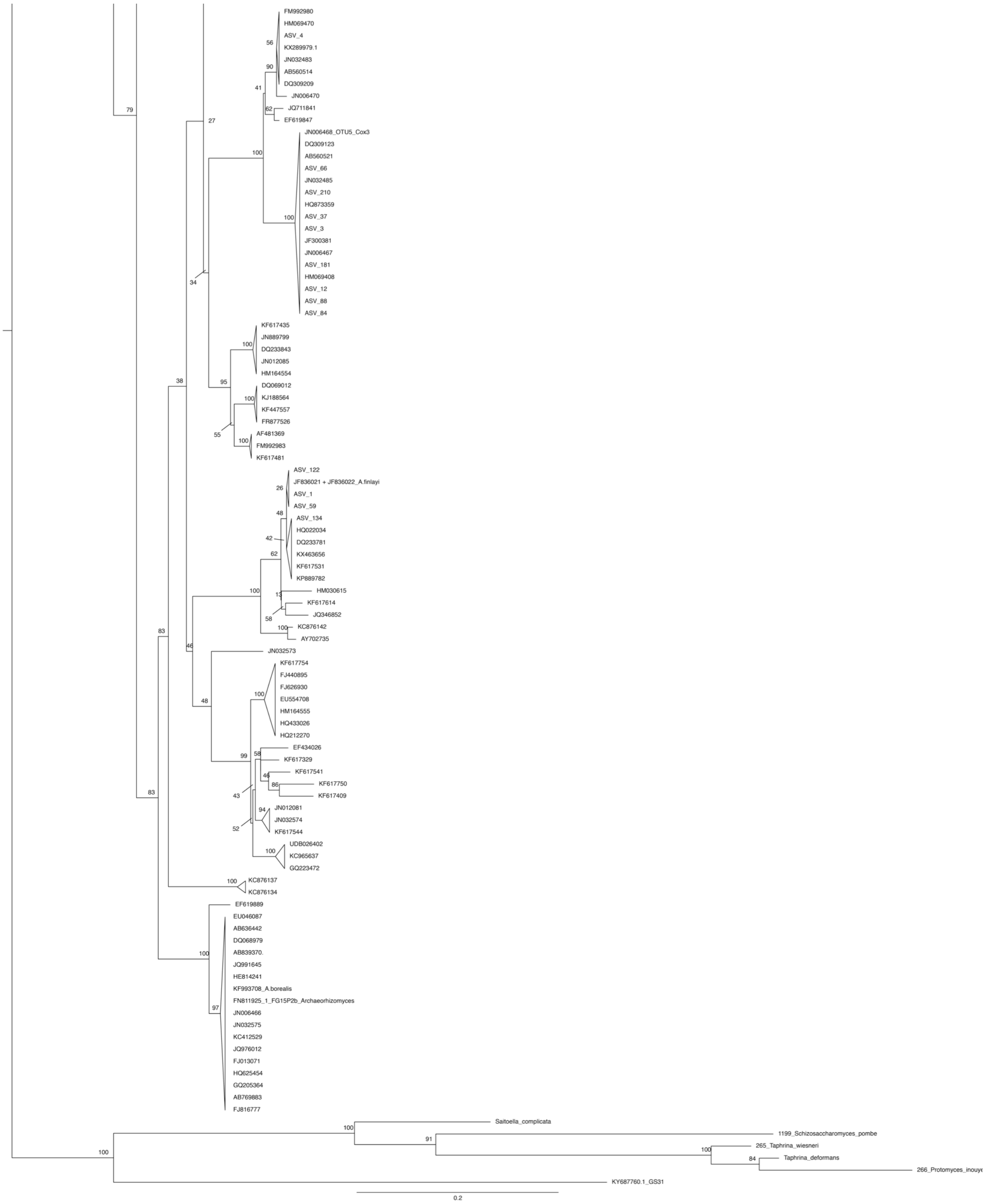

Figure S10

Maximum likelihood tree including all long read ASVs from the current study and publicly available environmental sequences of *Archaeorhizomycetes* covering at least two of the rDNA regions ITS1, ITS2 and LSU. The tree is limited to environmental sequences that cluster on well supported basal nodes with long read ASV sequences from the current study. Nodes represent the 76 species hypothesis (SH) based on Maximum likelihood solutions in PTP and are annotated with sequence names also detailed in Supplementary data file 6. Bootstrap supports are calculated from 1000 iterations. Tree files are available at https://doi.org/10.17605/OSF.IO/96DKZ

Supplementary methods

Method S1. Field site, soil sampling and isolation efforts

Soil samples were collected in mid-October 2013 from Ivantjärnsheden field station close to Jädraås (60°49'N, 16°30'E, altitude 185 m), a well-documented field site in central Sweden (Persson, 1980) with *Pinus sylvestris* L. overstory and an understory of ericaceous dwarf shrubs [*Calluna vulgaris* (L.) Hull and *Vaccinium vitis-ideae* L.] and mosses [*Dicranum majus* Turner and *Pleurozium schreberi* (Bridel) Mitten]. The stand was naturally regenerated after tree felling in 1957 and thinned before the 1974 onset of an experimental field study [experiment Ih2 (9802)] (Axelsson & Bråkenhielm, 1980) (Fig. S1). Between 1974 and 1990, 30 x 30 m plots received one of four different treatments: irrigation and fertilization (IF), irrigation (I), un-manipulated control (UM), as well as clear cut plots (CC). The clear-cut was part of a second study following the initial experiment and represented one plot from each previous treatment. Forest has since started to regenerate in CC plots.

To account for small-scale variability in soil fungal communities, we collected five soil cores (5 cm diameter and 15 cm deep) in each plot after visually dividing the plot into four quadrants; one core each were taken from the middle of each quadrant and from the middle of the plot after peeling back the top shrub and moss layer (incl. most of the litter layer). Soil cores were separated into visually distinct podzol soil layers: organic soil (O, approximately 0-5 cm depth), mineral elluvial soil (E, 5-8 cm) and mineral illuvial soil (B, 8-15 cm), before pooling the layers for each plot. We sampled four types of plots: three treatments (I: 11, 16, 17; IF: 4, 12, 13; O: 21, 23, 24) as well as the clear cuts (CC: 1, 2, 3) (Fig. S1, Table S1). This sampling rendered a total of 36 soil samples that were separately homogenized in Ziploc bags before separating a 15 mL sample from each that was transported back to the laboratory on ice and stored at -20°C.

An extensive culturing effort was performed during summer and fall of 2013 and 2014, attempting to isolate species in *Archaeorhizomycetes* from Ivantjärnsheden field station. New soil samples were collected for isolations in 2014. The isolation protocol was based on previous successful isolations of *Archaeorhizomycetes* (Grelet et al., 2010; Menkis et al., 2014; Rosling et al., 2011). Roots were separated from soil samples collected as described above. Under a stereomicroscope, healthy and dead root tips about 1 cm long were selected, and superficially disinfected in 30 % peroxide for 30 s and rinsed in sterile, deionized water for 2 min. Then, root tips were cut in ~ 2 mm long pieces and placed in 10 cm diameter petri dishes containing modified Melin Norkrans media (MMN) (Marx, 1969) with half strength of glucose. Up to 20 root fragments were placed in each dish and incubated up to five months in darkness at room temperature. Since available *Archaeorhizomycetes* cultures are slow-growing, all fungal colonies that grew from the root fragment during the first three months were discarded by cutting them out of the plate using a sterile scalpel. Fungal colonies emerging from the root tips after this period were sub-cultivated on new dishes with the same MMN media. Days after plating were recorded for all transfers. Approximately 2,000 root tips were surface-sterilized and plated, resulting in just over 160 cultures somewhat resembling those of *A. borealis* or *A. finlayi*. DNA was extracted from these for amplification of the rDNA ITS and LSU region, using the forward primers ITS1 (White et al., 1990) and reverse LR3 (Hopple Jr & Vilgalys, 1994), and then amplified and sequenced by Sanger technology, also including the reverse primer ITS4

(Gardes & Bruns, 1993). The 98 usable sequences generated were assigned to SH in UNITE (Kõljalg et al., 2013) and BLASTed against GenBank (Altschul et al., 1990). None of the sequences matched *Archaeorhizomycetes*, but many were identified as root associated fungi in the genera *Coemansia, Meliniomyces,* and *Phialocephala* (among others). The sequences were deposited in GenBank (Sayers et al., 2020), with accession numbers MH843963–MH844060.

Method S2. Soil DNA extraction

From each composite soil sample, two sub-samples of approximately 0.5 g wet weight were collected into separate 2.0 mL microtube containing 750 µL of lysis buffer (Xpedition™ Soil/Fecal DNA miniprep, Zymo Research Corporation, Irvine, California, USA). Total soil DNA extraction was performed after homogenization for 30 s using a TerraLyser™ (Zymo Research Corporation), following the manufacturer's protocol. DNA concentration and integrity were verified by 0.8 % agarose gel electrophoresis on 0.5 % Tris Acetate-EDTA buffer (Sigma-Aldrich, St. Louis, Missouri, USA) stained with 1 × GelRed™ (Biotium Inc., Hayward, California, USA).

Method S3. Generating the "phylogenetic" sequence dataset

Approximately 1,500 bp of the rDNA ITS and LSU region was amplified from all soil DNA extracts using the primer set ITS1F (Gardes & Bruns, 1993) and LR5 (Hopple Jr & Vilgalys, 1994), with Phusion High-Fidelity DNA polymerase (Thermo Fisher Scientific, Waltham, Massachusetts, USA). We ran a thermo-cycling protocol as follows: an initial denaturation at 95 °C for 10 min followed by 30 cycles of denaturation at 95 °C for 45 s, annealing at 58 °C for 45 s, and elongation at 72 °C for 90 s, with a final elongation at 72 °C for 10 min. A total of 5, 8 and 3 samples successfully amplified by PCR for O, E and B horizons, respectively (Table S1). PCR products from the separate soil horizons were pooled and quantified by Nanodrop 2000C (ThermoScientific, Waltham US), and gel electrophoresis was performed to generate three amplicon libraries (SwO, SwE and SwB) for sequencing at SciLifeLab/NGI (Uppsala, Sweden) on a PacBio RS II system (Pacific Biosciences, Menlo Park, CA, USA). Sequences were delivered to us as error-corrected FASTQ files. Raw reads for the current study are available in ENA (samples ERS3508481- ERS3508483).

The "phylogenetic" sequences dataset was filtered in four steps for downstream phylogenetic analysis of the *Archaeorhizomycetes* diversity at the site. First, the raw sequence reads were filtered and trimmed using the tool cutadapt (version 1.18) (Martin, 2011) to keep only reads with both primers present, and to remove the actual primer sequences from the reads. Amplicons sequenced in reverse, were reverse complemented before continuing the analyses. Secondly, a quality-controlled, long read sequence dataset of all amplified sequence variants (ASVs) was generated using DADA2 (version 1.9.3) (Callahan et al., 2016). Default parameters were used for filtering the reads, but discarding sequences with more than 12 "expected errors" (maxEE=12). In addition, sequences shorter than 50 bp and longer than 3,000 bp were removed as spurious. The option 'pooled' was used both for denoising and chimera detection, in order to consider the sequences in all three samples/data sets together. For chimera removal, the option 'AllowOneOff' was used to allow for one mismatch or indel. Thirdly, the tool ITSx (version 1.1-beta) (Bengtsson-Palme et al., 2013) was used to identify different regions of rDNA within each ASV. Seven of

the ASVs were considered incomplete reads and removed from further analysis because no ITS2 region was detected. BLASTing of these sequences suggest that they represent protists. For the remaining ASVs, taxonomy was predicted with the ITS2 region using the SINTAX classifier (Edgar, 2010) as implemented in VSEARCH (version 2.10.4) (Rognes et al. 2016), and the USEARCH/UTAX reference dataset (version 8.0) (UNITE Community, 2019), available from the UNITE database (Kõljalg et al., 2013). This reference dataset was customized by replacing unassigned species level taxonomy with UNITE SH when available. Geneious (version 11.1.4) (Kearse et al., 2012) was used to align the LSU region of the 276 ASVs with the LSU sequences of *A. finlayi* (JF836022), *A. borealis* (KF993708) and the uncultured lineage GS31 (KY687760) which represents a sister class of *Archaeorhizomycetes* (Tedersoo et al., 2017). The Geneious alignment algorithm was set to the Global alignment with free end gaps option, a cost matrix of 65% similarity (5.0/-4.0) with gap open penalty 12 and gap extension penalty 3. The alignment was visually inspected, and three ASVs were removed because two were truncated (ASV_279 and ASV_196) and one (ASV_280) was chimeric with a possible protist sequence and represent 5 reads.

A final alignment across 1,186bp from 273 ASV and 3 reference sequences was uploaded in the CIPRES portal (Miller et al., 2010) and a maximum likelihood tree was built using the online version of RAxML XSED2 (version 8) (Stamatakis, 2014). The GTRGAMMA model following recommendations on parametrization in (Kelchner & Thomas, 2007), and 1000 iterations for the calculation of bootstrap support. The tree was visualized in FigTree (version 1.4.4) and rooted with two Rozellomycota sequences (ASV_229 and ASV_237). Taxonomy predictions with a confidence value of 0.8 or higher using class when available, or else phyla or domain was added to the ASV name in the tree file using a customized script. Forty-two ASVs representing *Archaeorhizomycetes* were identified as those forming a well-supported clade together with *A. borealis* and *A. finlayi,* and distinct from GS31 (Fig. S2). The full-length sequences of these 42 ASVs were aligned in Geneious, as described above, and a ML tree was generated in the CIPRES portal as described above, and ASVs on long branches were visually inspected in the alignment. This procedure identified ASV_135 as a chimera formed from parent sequences ASV106 and ASV8, and was removed from the dataset. With these steps, we generated a high quality *Archaeorhizomycetes* rDNA sequence dataset consisting of 41 ASVs ranging in length from 1,373-1,801bp and representing 52,274 reads (Fig. S2, Table S2, S3). In the end, 272 ASV sequences, together with the most specific taxonomy level with a confidence value of 0.8 or above were published in GBIF (http://doi.org/10.15468/8zymuf) and are available in UNITE with accession numbers UDB0779092- UDB0779901.

Method S4. Generating the "ecological" sequence dataset

The ITS2 region of the rDNA genes was amplified using primers gITS7 *forward* (Ihrmark et al., 2012) and modified ITS4m *reverse* (Rosling et al., 2016) (a 1:1 mixture of ITS4 (White et al., 1990) and a modified version of it: 5'–TCCTC**GC**CTTATTGATATGC–3'), with both primers containing adequate barcode sequences for single-ended sequencing (Table S1). Modifications on the reverse primer ITS4 were included to reduce its known bias against the class *Archaeorhizomycetes* (Schadt & Rosling, 2015). PCR was performed in a final volume of 20 µL reactions with 10-20 ng DNA, 1 × SSoAdvanced^{TM} Universal SYBR® Green Supermix (Bio-Rad Laboratories, Hercules, California, USA), and 0.8 nM of each primer. PCR amplifications were carried out on a CFR96 Touch^{TM}Real/Time PCR Detection system (Bio-

Rad Laboratories) with 10 min pre-denaturation at 95 °C, 1 min DNA denaturation at 95 °C, 45 s annealing at 50, 54 and 58 °C (performed in separate tubes) to compensate for primer binding bias (Schmidt et al., 2013), 50 s of extension at 72 °C and 3 min final extension at 72 °C. Monitoring amplification by qPCR allowed us to adjust the number of cycles between 23-27 for each plate, in order to ensure that amplification did not plateau and to minimize chimera formation. All reactions were carried out in parallel on the duplicate DNA extracts from each sample, combining all six PCR products before purification using the ZR-96 DNA Clean & Concentrator™-5 (Zymo Research Corporation). Duplicate quantification of PCR products was performed using the Quant-iT™ PicoGreen® dsDNA Assay Kit (Life Technologies Corporation, Carlsbad, California, USA) on a TECAN F500 microplate reader and the band purity and length were checked by electrophoresis in 2 % agarose gel 0.5 × TAE buffer. A sequencing library was prepared by pooling 35 ng PCR products (when available) from each sample including both negative and positive template controls, loaded onto a 318 chip for PGM Ion Torrent sequencing technology (Life Technologies Corporation, Carlsbad, CA, US), and sequenced at SciLifeLab/NGI (Uppsala, Sweden). The current study was sequenced on the same IonTorrent chip as four other studies with samples from different ecosystems including forest soils across Puerto Rico (Urbina et al., 2016), mixed deciduous forest in Indiana, US (Rosling et al., 2016) and two un-published datasets from west Africa (Benin and Burkina Faso) and central Asia (India and Kyrgyzstan). The 4,805,942 raw sequence reads were demultiplexed by the sequencing facility and provided as 96 FASTQ files. Raw reads for the current study are available in ENA (samples ERS4600640-ERS4600675).

The software package DADA2 (version 1.14.0) (Callahan et al., 2016) for R (version 3.6.1) (R Core Team, 2019) was used to quality filter the raw reads and infer ASVs. Prior to ASV inference, primer and barcode sequences were trimmed from the reads using cutadapt (version 2.6 with Python version 3.7.5) (Martin, 2011), allowing for up to two mismatches, and requiring detection of both forward and reverse primers, as well as a minimum primer/read overlap of 10 bp. After primer trimming, 1,905,188 reads (39.6% of raw reads) were retained. The `filterAndTrim` function of the DADA2 package was then used to trim the first 15 bp (`trimLeft = 15`), truncate the reads at any quality score of two (`tuncQ = 2`), and filter out reads that are less than 75 bp in length (`minLen = 75`) or have more than five expected errors (`maxEE = 5`). Next, error learning and denoising (via the `learnErrors` and `dada` functions, respectively) of the reads were done using default parameters, except that a homopolymer gap penalty of 1 was specified for the denoising step (`HOMOPOLYMER_GAP_PENALTY = -1`), and the alignment band size was increased to 32 for both error learning and denoising steps (`BAND_SIZE = 32`). All 96 samples from the chip were pooled for ASV inference (`pool = TRUE`) and `removeBimeraDenovo` function was used to detect and remove chimeras (`method = “pooled”`) and allowing for a one-off mismatch (`allowOneOff = TRUE), resulting in a total of 4,822 ASVs, representing 1,804,814 reads (37.6% of raw reads). For clarity, ASVs generated from IonTorrent data are called itASVs throughout the text.

Two methods were used to identify itASVs as putative *Archaeorhizomycetes* sequences. In the first method, taxonomy was predicted using a bootstrap support cutoff of 0.8 for all itASVs using the SINTAX classifier in USEARCH (v11.0.667_i86osx32) (Edgar, 2010) with a modified version of the UNITE USEARCH/UTAX reference dataset for all Eukaryotes (version 8.0) (UNITE Community, 2019). Since the SINTAX classifier cannot handle blank fields in taxonomy strings, the UNITE reference dataset was modified to replace blank fields with ‘<lowest_name_ided>_<rank>_Incertae_sedis’, where <lowest_name_ided> is the

identity of the lowest identified supra-taxonomic rank, and <rank> is the taxonomic rank of the blank field, using the custom python script add_Incertae_sedis.py. These taxonomy predictions were also used to detect non-fungal sequences (based on a 0.8 bootstrap cutoff). In the second method, itASVs were BLASTed against a reference dataset consisting of the 41 *Archaeorhizomycetes* ASVs from the phylogenetic dataset, plus five sequences of the undescribed class-level lineage GS31 (GenBank Accession numbers KY687669, KY687744, KY687760, KY687776, and KY687785) (Tedersoo et al., 2017), using the blastn command line tool (version 2.9.0) (Camacho et al., 2009) with a minimum of 70% query coverage per high-scoring sepment pair (`-qcov_hsp_perc 70`). An itASV was identified as putatively *Archaeorhizomycetes* if it was detected via either method (i.e., any itASV with a blast hit to one of the *Archaeorhizomycetes* ASVs of the phylogenetic dataset or with a taxonomic prediction to *Archaeorhizomycetes*). A total of 182 and 282 itASVs were identified across the 96 samples via the SINTAX method and the BLAST method, respectively, and all 182 itASVs detected via the SINTAX method were also identified via the BLAST method.

After removing non-fungal itASVs as well as the sequence from the positive control DNA (itASV_1), the dataset consisted of 4,461 itASVs and 1,680,044 reads, of which 282 itASVs/425,349 reads were putatively *Archaeorhizomycetes* (25.3% of the fungal reads) across the entire sequencing run. The `metaMDS` function from the R package vegan (version 2.5-6) (Oksanen et al., 2013) was used to conduct an nMDS ordination of the complete itASV occurrence matrix across all samples (Fig. S3). The ordination was based on a distance matrix, calculated after transforming the matrix to per-sample relative abundances using the Bray-Curtis dissimilarity index, and a maximum of 200 random starts was specified. The results indicate that sample 24B may suffer from tag-switching with one of the samples from a different study, however we still choose to include this sample in all downstream analyses, since only seven rare *Archaeorhizomycetes* itASV were unique to sample 24B, and therefore the *Archaeorhizomycetes* community was not notably different from the other Jädraås samples. Finally, the "ecological" dataset (an itASV count per sample matrix for the Jädraås samples) was generated by removing all samples from other studies and positive/negative controls. Further, itASVs occurring only once across the 36 Jädraås samples were filtered out. This "ecological" dataset consisted of 1,664 itASVs (619,176 reads) (Supplementary datafile 1), sequences are published in GenBank (accession numbers MT926458 - MT928121), and 123 of these itASVs (233,667 reads; 37.7% of Jädraås fungal reads) were putatively *Archaeorhizomycetes* (Table S4, Supplementary datafile 2).

Method S5. Delimiting phylogenetic species hypotheses

<u>Phylogenetic species recognition</u>

We generated an alignment with the 123 itASVs from the "ecological" dataset and the 41 ASVs from the "phylogenetic" dataset including reference rDNA sequences of *A. borealis* (KF993708), *A. finlayi* (JF836021 and JF836022) and the uncultured lineage GS31 (KY687760) as well as several 5.8S and LSU rDNA sequences representing outgroup taxa in *Taphrinomycotina* (*Saitoella complicata* Goto, Sugiy., Hamam. & Komag. (AY548296), *Schizosaccharomyces pombe* Lindner (EU916982), *Taphrina deformans* (Berk.) Tul. (DQ470973) and *Taphrina wiesneri* (Ráthay) Mix (NG_027620)) using Geneious as above. To avoid duplication of data, we identified and removed 25 itASVs that were identical to the ITS2 regions of a long read ASV in the alignment (Supplementary datafile 3). A ML tree was inferred from the final alignment with 146 sequences (including 41 ASVs covering ITS and

LSU and 98 itASVs covering the ITS2) as described above. SHs were delimited using both Bayesian and Maximum likelihood implementations of the Poisson tree processes (PTP) model, using the online web server (Zhang et al., 2013) (Supplementary datafiles 4). FigTree v1.4.4 was used to visualize the resulting tree, and Maximum likelihood solution SHs were collapsed to their stem node for display. Only SHs supported by long reads were further analyzed for niche distribution. All alignments and trees are made available in TreeBASE, Study ID S26320.

Analysis of relative sequence read abundance and distribution of *Archaeorhizomycetes*
A 2-way ANOVA (function aov in R 3.4.1) (R Core Team, 2019) was used to test for general trends in total fungal reads, number of *Archaeorhizomycetes* itASVs, number of *Archaeorhizomycetes* SHs, and relative abundance of *Archaeorhizomycetes* across soil horizons and treatments administered in previous studies (Table S5, Supplementary datafile 5). Terms were added sequentially and post-hoc Tukey test with HSD p-value correction for multiple comparisons were performed when significant effects were detected. The overall *Archaeorhizomycetes* community composition in relation to soil horizon was visualized using nMDS ordination (metaMDS function in the vegan R package) based on the relative abundance of the 68 delimited *Archaeorhizomycetes* SHs using the default Bray-Curtis distance. Two dimensions were assigned to the analysis with a maximum of 100 random starts. The significance of soil horizon, plot and treatments in shaping the *Archaeorhizomycetes* community composition was subsequently tested using permutational multivariate analysis of variance (PERMANOVA) implemented in the anova.cca function in the vegan R package (Oksanen et al., 2013) (Table S6). This analysis was based on relative abundance of the nine SHs represented by long read, and 999 permutations were performed. We also tested for soil horizon specificity of the nine SH using a Kruskal-Wallis test in R (kruskal.test function in R version 3.4.1) (R Core Team, 2019). The multiple comparisons *p*-values were corrected with the Benjamini & Hochberg-method (Table S7).

IonTorrent sequencing generated 4.5 times more reads than PacBio sequencing and we thus expected the “ecological” dataset to represent a more exhaustive sampling compared to the phylogenetic dataset. We used the “ecological” dataset to estimate how much of the *Archaeorhizomycetes* species richness and abundance was represented by the “phylogenetic” dataset, by calculating the proportion of SHs including long read ASVs out of all SHs (Fig. 1, Table S4). Read abundance of SHs in the “ecological” dataset was used to estimate the detection limit of the “phylogenetic” dataset by determining the rarest SH including long read ASVs. However, detection limits are not absolute since not all soil samples were included in the “phylogenetic” dataset (Table S1).

Ecological species recognition
Based on the phylogenetic analysis of SHs detected at the site, we selected two pairs of sister SHs (SH_2 vs. SH_3 and SH_7 vs. SH_8) that were supported by long reads from the “phylogenetic” dataset and frequently observed in the “ecological” dataset (Fig. S4, Table S8). We tested these pairs for niche-specific distribution using relative abundance in the “ecological” dataset with soil horizons as operationally identified niches. Orthogonal contrasts for each pair were given as explanatory variables against soil horizon relative abundances in the statistical model, which was run with 200 permutations (Table S9).

Method S6. Placement of SHs with published sequences

To place *Archaeorhizomycetes* SHs from the current study in a larger phylogenetic context, an alignment that included publicly available environmental sequences previously identified as belonging to the *Archaeorhizomycetes* (Menkis et al., 2014), as well as new sequences affiliated with the class as identified by BLAST search in UNITE (Altschul et al., 1990). Environmental sequences were included if they covered at least two of the three rDNA regions ITS1, ITS2 or LSU. Duplicate sequences from individual studies were excluded. Sequences were aligned including the outgroup previously described and visually inspected using the Geneious software package (version 11.1.4) (Kearse et al., 2012) to remove suspected chimeras. The alignment was truncated after the LSU D3 region where some sequences had long inserts (Holst-Jensen et al., 1999) that was not covered by most previously published sequences. Further, insertions present in only two or less sequences were deleted because these positions often originated in previously published sequences where we had no mean to control for sequencing errors. Maximum likelihood trees were built using the online version of RAxML XSED2 (version 8) (Stamatakis, 2014) in CIPRES portal (Miller et al., 2010), specifying a GTRGAMMA model and 1000 iterations for the calculation of bootstrap support. To focus the analysis on SHs from the current study, a series of alignments and trees were generated through a process of stepwise removal of published sequences that separated on deep nodes without sequences generated in the current study. The final alignment included a total of 172 *Archaeorhizomycetes* sequences in addition to the 41 ASVs generated in the current study and six outgroup sequences (alignment and tree are available in TreeBASE Study S26320). SHs were delimitated across the tree using the bPTP portal as above and referred to as global SHs and visualized in TreeView by collapsing nodes corresponding to SHs according to the maximum likelihood solution. All included *Archaeorhizomycetes* sequences were mapped to UNITE species hypotheses at 98.5% by massBLAST of their ITS region (Supplementary datafile 6). The generated tree allowed us to evaluate the robustness of phylogenetic species delimitation in our local dataset and to visualize global sister clade relationships. Further, the larger *Archaeorhizomycetes* alignment was used to visually inspect and identify diagnostic sequences regions in both the ITS1 and ITS2 region for two novel species first hypothesized as SH_7 and SH_8 (Fig. 1).

Supplementary references

Altschul, S. F., Gish, W., Miller, W., Myers, E. W., & Lipman, D. J. (1990). Basic local alignment search tool. Journal of molecular biology, 215(3), 403-410.

Axelsson, B., & Bråkenhielm, S. (1980). Investigation sites of the Swedish coniferous forest project - biological and physiological features. In T. Persson (Ed.), Structure and Function of Northern Coniferous Forests - An Ecosystem Study (pp. 25 - 64). Berlings.

Bengtsson-Palme, J., Ryberg, M., Hartmann, M., Branco, S., Wang, Z., Godhe, A., De Wit, P., Sánchez-García, M., Ebersberger, I., & de Sousa, F. (2013). Improved software detection and extraction of ITS1 and ITS 2 from ribosomal ITS sequences of fungi and other eukaryotes for analysis of environmental sequencing data. Methods in ecology and evolution, 4(10), 914-919.

Callahan, B. J., McMurdie, P. J., Rosen, M. J., Han, A. W., Johnson, A. J. A., & Holmes, S. P. (2016). DADA2: high-resolution sample inference from Illumina amplicon data. Nature methods, 13(7), 581.

Camacho, C., Coulouris, G., Avagyan, V., Ma, N., Papadopoulos, J., Bealer, K., & Madden, T. L. (2009). BLAST+: architecture and applications. BMC bioinformatics, 10(1), 421.

Edgar, R. C. (2010). Search and clustering orders of magnitude faster than BLAST. Bioinformatics, 26(19), 2460-2461.

Gardes, M., & Bruns, T. D. (1993). ITS primers with enhanced specificity for basidiomycetes-application to the identification of mycorrhizae and rusts. Molecular Ecology, 2(2), 113-118.

Grelet, G. A., Johnson, D., Vrålstad, T., Alexander, I. J., & Anderson, I. C. (2010). New insights into the mycorrhizal Rhizoscyphus ericae aggregate: spatial structure and co-colonization of ectomycorrhizal and ericoid roots. New Phytologist, 188(1), 210-222.

Holst-Jensen, A., Vaage, M., Schumacher, T., & Johansen, S. (1999). Structural characteristics and possible horizontal transfer of group I introns between closely related plant pathogenic fungi. Molecular biology and evolution, 16(1), 114-126. https://doi.org/10.1093/oxfordjournals.molbev.a026031

Hopple Jr, J. S., & Vilgalys, R. (1994). Phylogenetic relationships among coprinoid taxa and allies based on data from restriction site mapping of nuclear rDNA. Mycologia, 86(1), 96-107.

Ihrmark, K., Bödeker, I., Cruz-Martinez, K., Friberg, H., Kubartova, A., Schenck, J., Strid, Y., Stenlid, J., Brandström-Durling, M., & Clemmensen, K. E. (2012). New primers to amplify the fungal ITS2 region–evaluation by 454-sequencing of artificial and natural communities. Fems Microbiology Ecology, 82(3), 666-677.

Kearse, M., Moir, R., Wilson, A., Stones-Havas, S., Cheung, M., Sturrock, S., Buxton, S., Cooper, A., Markowitz, S., & Duran, C. (2012). Geneious Basic: an integrated and extendable desktop software platform for the organization and analysis of sequence data. Bioinformatics, 28(12), 1647-1649.

Kelchner, S. A., & Thomas, M. A. (2007). Model use in phylogenetics: nine key questions. Trends in Ecology & Evolution, 22(2), 87-94.

Kõljalg, U., Nilsson, R. H., Abarenkov, K., Tedersoo, L., Taylor, A. F., Bahram, M., Bates, S. T., Bruns, T. D., Bengtsson-Palme, J., & Callaghan, T. M. (2013). Towards a unified paradigm for sequence-based identification of fungi. Molecular Ecology, 22(21), 5271-5277.

Martin, M. (2011). Cutadapt removes adapter sequences from high-throughput sequencing reads. EMBnet. journal, 17(1), 10-12.

Marx, D. H. (1969). Influence of ectotrophic mycorrhizal fungi on resistance or pine roots to pathogenic infections. I Antagonisum of mycorrhizal fungi to root pathogenic fungi and soil bacteria. Phytopathology, 59, 153-163.

Menkis, A., Urbina, H., James, T. Y., & Rosling, A. (2014, 2014-Dec). Archaeorhizomyces borealis sp. nov. and a sequence-based classification of related soil fungal species. Fungal biology, 118(12), 943-955. https://doi.org/10.1016/j.funbio.2014.08.005

Miller, M. A., Pfeiffer, W., & Schwartz, T. (2010). Creating the CIPRES Science Gateway for inference of large phylogenetic trees. 2010 gateway computing environments workshop (GCE),

Oksanen, J., Blanchet, F. G., Kindt, R., Legendre, P., Minchin, P. R., O'hara, R., Simpson, G. L., Solymos, P., Stevens, M. H. H., & Wagner, H. (2013). Package 'vegan'. Community ecology package, version, 2(9), 1-295.

Persson, T. (1980). *Structure and function of northern coniferous forests; an ecosystem study* (Vol. 32). Arlöv, Sweden: Berlings.

R Development Core Team (2019). R: A Language and Environment for Statistical Computing. Vienna, Austria: R Foundation for Statistical Computing.

Rosling, A., Cox, F., Cruz-Martinez, K., Ihrmark, K., Grelet, G.-A., Lindahl, B. D., Menkis, A., & James, T. Y. (2011, Aug 12). Archaeorhizomycetes: Unearthing an Ancient Class of Ubiquitous Soil Fungi. Science, 333(6044), 876-879. https://doi.org/10.1126/science.1206958

Rosling, A., Midgley, M. G., Cheeke, T., Urbina, H., Fransson, P., & Phillips, R. P. (2016, FEB 2016). Phosphorus cycling in deciduous forest soil differs between stands dominated by ecto- and arbuscular mycorrhizal trees. New Phytologist, 209(3), 1184-1195. https://doi.org/10.1111/nph.13720

Sayers, E. W., Cavanaugh, M., Clark, K., Ostell, J., Pruitt, K. D., & Karsch-Mizrachi, I. (2020). GenBank. Nucleic acids research, 48(D1), D84-D86.

Schadt, C. W., & Rosling, A. (2015, 2015-Jun-26). FUNGAL BIOGEOGRAPHY. Comment on "Global diversity and geography of soil fungi". Science (New York, N.Y.), 348(6242), 1438. https://doi.org/10.1126/science.aaa4269

Schmidt, P.-A., Bálint, M., Greshake, B., Bandow, C., Römbke, J., & Schmitt, I. (2013). Illumina metabarcoding of a soil fungal community. Soil Biology and Biochemistry, 65, 128-132.

Stamatakis, A. (2014). RAxML version 8: a tool for phylogenetic analysis and post-analysis of large phylogenies. Bioinformatics, 30(9), 1312-1313.

Tedersoo, L., Bahram, M., Puusepp, R., Nilsson, R. H., & James, T. Y. (2017). Novel soil-inhabiting clades fill gaps in the fungal tree of life. Microbiome, 5(1), 42.

UNITE Community. (2019). Unite usearch/utax release for eukaryotes. Version 18.11.2018.

Urbina, H., Scofield, D. G., Cafaro, M., & Rosling, A. (2016, MAY 2016). DNA-metabarcoding uncovers the diversity of soil-inhabiting fungi in the tropical island of Puerto Rico. Mycoscience, 57(3), 217-227. https://doi.org/10.1016/j.myc.2016.02.001

White, T. J., Bruns, T., Lee, S., & Taylor, J. (1990). Amplification and direct sequencing of fungal ribosomal RNA genes for phylogenetics. In PCR protocols: a guide to methods and applications (Innis, MA, Gelfand, DH, Sninsky, JJ, White, Thomas J ed., Vol. 18, pp. 315-322). Academic Press.

Zhang, J., Kapli, P., Pavlidis, P., & Stamatakis, A. (2013). A general species delimitation method with applications to phylogenetic placements. Bioinformatics, 29(22), 2869-2876.
